# Supplementary material for: Longitudinal metabolic profiling of women using selective serotonin reuptake inhibitors during pregnancy
Source: Metabolomics. 2025 Aug 21;21(5):123. doi: 10.1007/s11306-025-02334-z (PMC12370871; doi:10.1007/s11306-025-02334-z)
Supplement: Supplementary file 1 — Supplementary Material 1 [file 11306_2025_2334_MOESM1_ESM.pdf]

Online Supplementary information

LONGITUDINAL METABOLIC PROFILING OF WOMEN USING SELECTIVE SEROTONIN REUPTAKE INHIBITORS DURING PREGNANCY

Anna ITKONEN MSc.[1], Olli KÄRKKÄINEN DSc.[1], Heidi SAHLMAN PhD[1], Leea KESKI-NISULA MD, PhD[2,3], Jaana RYSÄ PhD[1]

[1] School of Pharmacy, University of Eastern Finland, Finland

[2] Institute of Clinical Medicine, School of Medicine, University of Eastern Finland, Finland

[3] Department of Obstetrics and Gynecology, Kuopio University Hospital, Finland

Table S1. Information on SSRI usage and indications for SSRIs.

SSRI use		
**SSRI**	**Dose (mg)**	**n (%)**
Citalopram	5–40	45 (36.8)
Escitalopram	2.5–20	34 (27.9)
Sertraline	25–200	31 (25.4)
Fluoxetine	5–60	6 (4.9)
Paroxetine	5–40	3 (2.5)
Fluvoxamine	150	1 (0.8)
Escitalopram, Fluoxetine	20, 40	1 (0.8)
Escitalopram, Sertraline	20, 100	1 (0.8)
**SSRI usage during pregnancy**		**n (%)**
Permanent		75 (61.5)
Discontinued during 1st trimester		10 (8.2)
Discontinued during 2nd trimester		7 (5.7)
Discontinued during 3rd trimester		18 (14.8)
Initiated during 1st trimester		2 (1.6)
Initiated during 2nd trimester		6 (4.9)
Initiated during 3rd trimester		4 (3.3)
**Indications for SSRIs[1]**		
**Indication**		**n (%)**
Depression		79 (64.8)
Panic disorder		23 (18.9)
Anxiety		12 (9.8)
Other (bipolar disorder, obsessive-compulsive disorder, depersonalization-derealization disorder, social anxiety disorder)		5 (4.1)

[1] n=119; SSRI = Selective serotonin reuptake inhibitor

Table S2. Wilcoxon rank-sum test of the difference between women using selective serotonin reuptake inhibitors (SSRIs) during pregnancy and control women. The difference in the variables has been analyzed at two timepoints (1st trimester and at delivery). Moreover, the effect of SSRIs on the pregnancy-driven change in the metabolome (delta) has been compared by Wilcoxon rank-sum test.

				1ST TRIMESTER (N=185)										DELIVERY (N=201)										CHANGE BETWEEN DELIVERY AND 1ST TRIMESTER (DELTA) (N=150)								
				CONTROL (N=115)				SSRI (N=70)						CONTROL (N=112)				SSRI (N=89)						CONTROL (N=110)				SSRI (N=40)				
GROUP	VARIABLE	ABBRE-VIATION	UNIT	MEDIAN	IQR	MEAN	SD	MEDIAN	IQR	MEAN	SD	r	p	MEDIAN	IQR	MEAN	SD	MEDIAN	IQR	MEAN	SD	r	p	MEDIAN	IQR	MEAN	SD	MEDIAN	IQR	MEAN	SD	r
AMINO ACIDS	Alanine	Ala	mmol/l	0.38	0.07	0.37	0.06	0.39	0.09	0.38	0.07	0.06	0.4978	0.45	0.13	0.47	0.11	0.46	0.10	0.47	0.08	0.04	0.6401	0.09	0.12	0.09	0.11	0.08	0.13	0.08	0.10	-0.08
AMINO ACIDS	Glutamine	Gln	mmol/l	0.54	0.08	0.54	0.06	0.52	0.09	0.53	0.07	-0.14	0.1239	0.48	0.11	0.50	0.10	0.48	0.10	0.48	0.08	-0.06	0.4455	-0.04	0.12	-0.04	0.09	-0.06	0.10	-0.06	0.09	-0.09
AMINO ACIDS	Glycine	Gly	mmol/l	0.22	0.05	0.21	0.04	0.21	0.06	0.20	0.05	-0.09	0.2955	0.22	0.07	0.22	0.05	0.20	0.07	0.21	0.05	-0.19	**0.0192**	0.00	0.06	0.01	0.05	0.01	0.05	0.01	0.05	0.00
AMINO ACIDS	Histidine	His	mmol/l	0.10	0.01	0.10	0.01	0.10	0.02	0.10	0.01	-0.06	0.4608	0.09	0.02	0.09	0.02	0.09	0.02	0.09	0.02	-0.13	0.1085	-0.01	0.03	-0.01	0.02	-0.01	0.03	-0.01	0.02	-0.04
AMINO ACIDS (AROMATIC)	Phenylalanine	Phe	mmol/l	0.08	0.02	0.08	0.01	0.08	0.02	0.07	0.02	-0.09	0.3061	0.09	0.03	0.09	0.02	0.09	0.02	0.09	0.01	-0.01	0.8980	0.01	0.02	0.01	0.02	0.01	0.03	0.01	0.02	0.05
AMINO ACIDS (AROMATIC)	Tyrosine	Tyr	mmol/l	0.06	0.02	0.06	0.02	0.06	0.02	0.06	0.02	-0.08	0.3451	0.05	0.02	0.05	0.01	0.05	0.01	0.05	0.01	0.00	0.9854	-0.01	0.03	-0.01	0.02	-0.01	0.02	-0.01	0.02	-0.01
AMINO ACIDS (BRANCHED-CHAIN)	Isoleucine	Ile	mmol/l	0.05	0.03	0.05	0.02	0.06	0.03	0.05	0.02	0.04	0.6153	0.04	0.01	0.04	0.01	0.04	0.01	0.04	0.01	0.07	0.3670	-0.01	0.02	-0.01	0.02	-0.01	0.03	-0.02	0.02	-0.04
AMINO ACIDS (BRANCHED-CHAIN)	Leucine	Leu	mmol/l	0.11	0.05	0.10	0.03	0.12	0.05	0.11	0.04	0.05	0.5916	0.09	0.02	0.09	0.02	0.09	0.02	0.09	0.02	0.07	0.4003	-0.02	0.05	-0.03	0.04	-0.03	0.05	-0.03	0.04	-0.01
AMINO ACIDS (BRANCHED-CHAIN)	Total concentration of branched-chain amino acids (leucine + isoleucine + valine)	Total BCAA	mmol/l	0.40	0.15	0.39	0.09	0.41	0.15	0.37	0.12	0.03	0.7118	0.31	0.07	0.32	0.06	0.31	0.07	0.32	0.06	0.06	0.4325	-0.08	0.14	-0.08	0.11	-0.07	0.15	-0.09	0.10	-0.03
AMINO ACIDS (BRANCHED-CHAIN)	Valine	Val	mmol/l	0.23	0.07	0.23	0.05	0.24	0.08	0.22	0.05	0.03	0.7437	0.18	0.04	0.19	0.03	0.19	0.04	0.19	0.03	0.07	0.3922	-0.04	0.08	-0.04	0.06	-0.04	0.07	-0.05	0.05	-0.04
APOLIPOPROTEINS	Apolipoprotein A1	ApoA1	g/l	1.83	0.29	1.85	0.21	1.78	0.30	1.82	0.24	-0.12	0.1590	2.06	0.41	2.07	0.33	2.04	0.41	2.03	0.31	-0.06	0.4981	0.24	0.37	0.24	0.30	0.24	0.37	0.19	0.26	-0.06
APOLIPOPROTEINS	Apolipoprotein B	ApoB	g/l	0.71	0.22	0.70	0.16	0.76	0.19	0.73	0.17	0.16	0.0720	1.33	0.42	1.33	0.34	1.27	0.45	1.31	0.34	-0.05	0.5571	0.59	0.35	0.62	0.30	0.53	0.38	0.52	0.26	-0.18
APOLIPOPROTEINS	Ratio of apolipoprotein B to apolipoprotein A1	ApoB/ApoA1	ratio	0.39	0.13	0.38	0.10	0.43	0.14	0.41	0.11	0.21	**0.0144**	0.63	0.25	0.67	0.24	0.63	0.22	0.66	0.19	0.01	0.9504	0.23	0.20	0.27	0.20	0.20	0.11	0.22	0.13	-0.15
CHOLESTEROLS	Clinical LDL cholesterol	Clinical LDL-C	mmol/l	2.20	0.89	2.14	0.57	2.36	0.65	2.31	0.63	0.13	0.1281	3.66	1.27	3.77	1.04	3.45	1.17	3.58	1.00	-0.09	0.2704	1.51	1.21	1.55	0.89	1.26	0.95	1.13	0.87	-0.23
CHOLESTEROLS	HDL cholesterol	HDL-C	mmol/l	1.81	0.40	1.89	0.26	1.71	0.40	1.73	0.31	-0.21	**0.0147**	1.89	0.49	1.91	0.41	1.82	0.56	1.84	0.41	-0.11	0.1720	0.11	0.45	0.11	0.36	0.11	0.44	0.06	0.32	-0.06
CHOLESTEROLS	LDL cholesterol	LDL-C	mmol/l	1.59	0.54	1.55	0.35	1.70	0.42	1.64	0.40	0.15	0.0795	2.58	0.72	2.64	0.63	2.45	0.73	2.55	0.62	-0.08	0.3046	1.03	0.72	1.05	0.55	0.88	0.62	0.79	0.53	-0.24
CHOLESTEROLS	Remnant cholesterol (non-HDL. non-LDL -cholesterol)	Remnant-C	mmol/l	1.33	0.40	1.33	0.33	1.41	0.41	1.32	0.37	0.11	0.2284	2.64	0.91	2.64	0.66	2.47	0.86	2.60	0.67	-0.06	0.4514	1.27	0.75	1.32	0.59	1.09	0.77	1.11	0.53	-0.19
CHOLESTEROLS	Total cholesterol	Total-C	mmol/l	4.72	0.95	4.68	0.73	4.83	1.01	4.63	0.89	0.04	0.6908	7.08	1.54	7.20	1.30	6.78	1.66	6.98	1.35	-0.11	0.1987	2.34	1.48	2.48	1.19	2.07	1.69	1.96	1.18	-0.21
CHOLESTEROLS	Total cholesterol minus HDL-C	non-HDL-C	mmol/l	2.91	0.97	2.87	0.66	3.11	0.82	2.96	0.75	0.13	0.1274	5.28	1.55	5.29	1.27	4.93	1.41	5.14	1.26	-0.07	0.3908	2.24	1.34	2.37	1.12	2.09	1.32	1.90	1.03	-0.22
CHOLESTEROLS	VLDL cholesterol	VLDL-C	mmol/l	0.48	0.24	0.46	0.17	0.55	0.27	0.52	0.20	0.20	**0.0254**	1.27	0.57	1.29	0.42	1.25	0.57	1.31	0.44	0.01	0.8807	0.77	0.49	0.81	0.38	0.69	0.50	0.72	0.33	-0.13
CHOLESTERYL ESTERS	Cholesteryl esters in HDL	HDL-CE	mmol/l	1.40	0.29	1.45	0.20	1.32	0.31	1.33	0.24	-0.22	**0.0132**	1.39	0.38	1.40	0.33	1.33	0.47	1.34	0.32	-0.12	0.1413	0.02	0.35	0.01	0.29	0.00	0.33	-0.02	0.25	-0.06
CHOLESTERYL ESTERS	Total esterified cholesterol	Total-CE	mmol/l	3.48	0.69	3.45	0.52	3.54	0.69	3.40	0.63	0.02	0.7782	5.05	1.10	5.14	0.91	4.86	1.21	4.96	0.95	-0.11	0.1644	1.60	1.08	1.66	0.84	1.34	1.20	1.28	0.84	-0.20
FATTY ACIDS	Degree of unsaturation	Unsaturation	degree	1.35	0.07	1.35	0.06	1.34	0.08	1.34	0.06	-0.04	0.6293	1.25	0.08	1.25	0.06	1.22	0.09	1.23	0.07	-0.20	**0.0140**	-0.10	0.08	-0.11	0.06	-0.11	0.09	-0.12	0.07	-0.10
FATTY ACIDS	Docosahexaenoic acid	DHA	mmol/l	0.33	0.08	0.32	0.06	0.33	0.06	0.32	0.07	0.06	0.4765	0.38	0.11	0.39	0.10	0.38	0.14	0.38	0.10	-0.03	0.7334	0.05	0.08	0.06	0.09	0.04	0.08	0.04	0.07	-0.16
FATTY ACIDS	Linoleic acid	LA	mmol/l	3.96	0.64	3.90	0.57	4.03	0.73	3.89	0.66	0.06	0.5086	5.48	1.11	5.58	0.98	5.45	1.41	5.52	0.99	-0.03	0.7006	1.50	0.91	1.64	0.90	1.44	1.23	1.38	0.85	-0.14
FATTY ACIDS	Monounsaturated fatty acids	MUFA	mmol/l	2.83	0.76	2.71	0.67	3.10	0.93	2.97	0.85	0.22	**0.0141**	5.53	1.67	5.49	1.28	5.50	1.93	5.83	1.55	0.11	0.1806	2.60	1.67	2.69	1.19	2.35	1.79	2.63	1.31	-0.05
FATTY ACIDS	Omega-3 fatty acids	Omega-3	mmol/l	0.63	0.24	0.62	0.18	0.67	0.24	0.64	0.20	0.14	0.1094	0.83	0.31	0.86	0.27	0.86	0.42	0.89	0.26	0.08	0.3270	0.20	0.23	0.22	0.24	0.14	0.22	0.18	0.18	-0.10
FATTY ACIDS	Omega-6 fatty acids	Omega-6	mmol/l	4.70	0.71	4.63	0.56	4.82	0.93	4.69	0.69	0.10	0.2702	6.36	1.13	6.49	0.97	6.43	1.57	6.46	1.00	-0.02	0.8157	1.77	1.00	1.80	0.90	1.47	1.25	1.51	0.86	-0.17
FATTY ACIDS	Polyunsaturated fatty acids	PUFA	mmol/l	5.33	0.86	5.20	0.68	5.49	1.23	5.36	0.85	0.11	0.1972	7.25	1.33	7.35	1.12	7.23	1.77	7.35	1.15	0.00	0.9543	1.96	1.20	2.02	1.06	1.65	1.58	1.69	0.95	-0.16
FATTY ACIDS	Saturated fatty acids	SFA	mmol/l	4.27	0.81	4.17	0.87	4.47	1.23	4.34	0.92	0.14	0.1003	7.61	2.07	7.71	1.51	7.98	1.86	8.11	1.69	0.11	0.1953	3.39	1.98	3.46	1.46	3.27	1.89	3.45	1.47	-0.01
FATTY ACIDS	Total fatty acids	Total-FA	mmol/l	12.43	2.22	12.14	2.11	13.06	3.28	12.70	2.48	0.16	0.0631	20.36	5.42	20.55	3.67	20.41	4.91	21.29	4.09	0.08	0.3517	7.91	4.60	8.18	3.46	7.79	4.63	7.77	3.42	-0.07
FATTY ACIDS (RATIOS	Ratio of docosahexaenoic acid to total fatty acids	DHA %	%	2.64	0.59	2.60	0.39	2.56	0.56	2.58	0.39	-0.08	0.3539	1.90	0.48	1.93	0.42	1.79	0.52	1.83	0.46	-0.15	0.0774	-0.72	0.46	-0.73	0.34	-0.84	0.51	-0.76	0.36	-0.01
FATTY ACIDS (RATIOS)	Ratio of linoleic acid to total fatty acids	LA %	%	32.07	3.27	32.22	2.73	31.06	3.40	31.12	2.56	-0.23	**0.0076**	27.64	3.88	27.36	3.04	26.15	4.24	26.17	3.15	-0.22	**0.0079**	-4.58	2.96	-4.74	3.06	-4.49	4.00	-4.78	3.04	0.00
FATTY ACIDS (RATIOS)	Ratio of monounsaturated fatty acids to total fatty acids	MUFA %	%	22.57	2.93	22.45	1.87	23.47	2.87	22.85	2.50	0.20	**0.0245**	26.45	2.75	26.51	2.06	27.20	3.66	27.10	2.74	0.14	0.0837	3.89	2.80	4.03	2.05	3.07	2.85	3.61	2.62	-0.14
FATTY ACIDS (RATIOS)	Ratio of omega-3 fatty acids to total fatty acids	Omega-3 %	%	5.03	1.62	4.96	1.01	5.12	1.14	5.07	1.00	0.06	0.4871	4.18	1.55	4.17	1.02	4.09	1.41	4.19	1.01	0.02	0.8119	-0.92	1.10	-0.91	0.82	-1.14	1.00	-1.06	0.70	-0.10
FATTY ACIDS (RATIOS)	Ratio of omega-6 fatty acids to omega-3 fatty acids	Omega-6/Omega-3	%	7.93	2.54	7.77	1.86	7.57	1.73	7.41	1.73	-0.12	0.1658	7.63	3.13	8.15	2.30	7.75	3.25	7.80	2.30	-0.09	0.2507	0.24	1.91	0.28	1.67	0.61	2.08	0.39	1.44	0.07
FATTY ACIDS (RATIOS)	Ratio of omega-6 fatty acids to total fatty acids	Omega-6 %	%	38.17	3.00	38.67	2.78	37.26	3.68	37.51	2.69	-0.22	**0.0112**	32.08	3.41	31.88	2.88	30.68	3.69	30.70	3.17	-0.24	**0.0042**	-6.44	3.61	-6.37	3.12	-6.43	4.73	-6.38	3.43	0.02

				1ST TRIMESTER (N=185)										DELIVERY (N=201)										CHANGE BETWEEN DELIVERY AND 1ST TRIMESTER (DELTA) (N=150)								
				CONTROL (N=115)				SSRI (N=70)						CONTROL (N=112)				SSRI (N=89)						CONTROL (N=110)				SSRI (N=40)				
GROUP	VARIABLE	ABBRE-VIATION	UNIT	MEDIAN	IQR	MEAN	SD	MEDIAN	IQR	MEAN	SD	r	p	MEDIAN	IQR	MEAN	SD	MEDIAN	IQR	MEAN	SD	r	p	MEDIAN	IQR	MEAN	SD	MEDIAN	IQR	MEAN	SD	r
FATTY ACIDS (RATIOS)	Ratio of polyunsaturated fatty acids to monounsaturated fatty acids	PUFA/MUFA	%	1.93	0.34	1.93	0.26	1.83	0.31	1.84	0.29	-0.20	**0.0200**	1.38	0.26	1.37	0.20	1.27	0.30	1.31	0.25	-0.21	**0.0123**	-0.59	0.32	-0.57	0.25	-0.50	0.37	-0.52	0.28	0.14
FATTY ACIDS (RATIOS)	Ratio of polyunsaturated fatty acids to total fatty acids	PUFA %	%	43.20	3.17	43.54	2.73	42.38	3.60	42.68	2.85	-0.18	**0.0408**	36.49	3.71	36.04	2.94	34.84	3.92	34.89	3.39	-0.25	**0.0027**	-7.18	3.25	-7.27	3.22	-7.18	5.73	-7.44	3.51	0.01
FATTY ACIDS (RATIOS)	Ratio of saturated fatty acids to total fatty acids	SFA %	%	34.22	1.67	33.88	1.87	34.15	2.22	34.15	1.61	0.01	0.9042	37.14	1.96	37.45	1.84	37.78	2.63	38.01	2.05	0.19	**0.0178**	3.14	2.00	3.24	2.21	3.51	2.98	3.83	2.28	0.10
FLUID BALANCE	Albumin	Albumin	g/l	39.13	2.71	38.97	2.94	38.65	4.65	38.60	3.07	-0.10	0.2593	28.86	3.22	28.88	2.57	28.64	4.47	28.15	3.27	-0.15	0.0718	-10.29	3.73	-10.31	3.27	-10.56	4.39	-10.89	3.45	-0.09
FLUID BALANCE	Creatinine[2,3]	Creatinine	µmol/l	53.38	9.96	52.65	6.88	54.32	10.02	54.35	7.61	0.08	0.3785	65.62	12.92	65.93	9.63	64.75	14.10	64.15	11.28	-0.10	0.2209	12.80	13.89	12.53	9.26	8.44	16.51	10.08	12.84	-0.13
FREE CHOLESTEROL	Free cholesterol in HDL	HDL-FC	mmol/l	0.41	0.11	0.43	0.07	0.39	0.08	0.40	0.08	-0.17	0.0596	0.50	0.13	0.51	0.09	0.49	0.11	0.50	0.10	-0.06	0.4426	0.09	0.11	0.10	0.09	0.08	0.12	0.09	0.08	-0.08
FREE CHOLESTEROL	Free cholesterol in LDL	LDL-FC	mmol/l	0.44	0.14	0.43	0.09	0.46	0.10	0.45	0.10	0.11	0.2072	0.65	0.19	0.67	0.16	0.61	0.19	0.63	0.16	-0.12	0.1348	0.21	0.18	0.23	0.14	0.17	0.19	0.15	0.15	-0.22
FREE CHOLESTEROL	Free cholesterol in VLDL	VLDL-FC	mmol/l	0.19	0.09	0.18	0.08	0.23	0.11	0.21	0.09	0.22	**0.0107**	0.53	0.26	0.54	0.18	0.54	0.26	0.56	0.20	0.05	0.5720	0.33	0.24	0.35	0.17	0.29	0.24	0.32	0.16	-0.09
FREE CHOLESTEROL	Total free cholesterol	Total-FC	mmol/l	1.24	0.29	1.23	0.22	1.28	0.30	1.22	0.25	0.06	0.4800	2.05	0.47	2.06	0.40	1.94	0.50	2.02	0.41	-0.08	0.3429	0.74	0.43	0.82	0.36	0.66	0.49	0.68	0.34	-0.20
GLYCOLYSIS RELATED METABOLITES	Citrate	Citrate	mmol/l	0.06	0.01	0.06	0.01	0.06	0.01	0.06	0.01	0.05	0.5343	0.08	0.02	0.08	0.01	0.08	0.02	0.09	0.02	0.14	0.0938	0.02	0.02	0.02	0.01	0.03	0.03	0.03	0.02	0.14
GLYCOLYSIS RELATED METABOLITES	Glucose	Glucose	mmol/l	4.72	0.93	4.62	0.86	5.03	0.88	4.80	0.85	0.24	**0.0067**	5.07	1.43	5.46	1.30	5.43	1.32	5.66	1.22	0.14	0.0782	0.59	1.48	0.76	1.40	0.75	1.54	0.63	1.75	-0.05
GLYCOLYSIS RELATED METABOLITES	Glycerol[4]	Glycerol	mmol/l	0.10	0.04	0.10	0.03	0.11	0.05	0.09	0.04	0.01	0.9025	0.15	0.05	0.15	0.04	0.16	0.05	0.16	0.05	0.12	0.1418	0.05	0.07	0.05	0.05	0.06	0.05	0.06	0.06	0.06
GLYCOLYSIS RELATED METABOLITES	Lactate[2]	Lactate	mmol/l	2.28	0.73	2.22	0.59	2.42	0.92	2.32	0.66	0.12	0.1884	3.08	1.30	3.27	1.11	3.21	1.18	3.34	0.94	0.06	0.4797	0.98	1.48	1.02	1.15	0.99	1.18	1.07	1.16	0.02
GLYCOLYSIS RELATED METABOLITES	Pyruvate	Pyruvate	mmol/l	0.05	0.04	0.05	0.03	0.06	0.05	0.05	0.04	0.10	0.2659	0.11	0.05	0.12	0.05	0.12	0.05	0.13	0.05	0.08	0.3442	0.06	0.06	0.07	0.06	0.06	0.08	0.07	0.08	-0.07
INFLAMMATION	Glycoprotein acetyls	GlycA	mmol/l	0.84	0.14	0.83	0.10	0.88	0.16	0.86	0.12	0.15	0.0963	1.00	0.12	1.01	0.10	1.06	0.14	1.05	0.12	0.23	**0.0044**	0.17	0.15	0.16	0.10	0.15	0.15	0.16	0.11	0.00
KETONE BODIES	3-Hydroxybutyrate	bOHbutyrate	mmol/l	0.04	0.02	0.03	0.04	0.04	0.03	0.03	0.05	0.04	0.6567	0.08	0.11	0.15	0.19	0.09	0.14	0.15	0.14	0.03	0.7593	0.05	0.11	0.11	0.19	0.06	0.12	0.11	0.16	0.06
KETONE BODIES	Acetate	Acetate	mmol/l	0.03	0.01	0.03	0.01	0.03	0.01	0.03	0.01	0.01	0.8863	0.02	0.01	0.03	0.01	0.02	0.01	0.03	0.01	-0.05	0.5786	0.00	0.02	0.00	0.02	-0.01	0.02	0.00	0.01	-0.11
KETONE BODIES	Acetoacetate	Acetoacetate	mmol/l	0.02	0.01	0.02	0.01	0.03	0.01	0.02	0.02	0.19	**0.0315**	0.04	0.04	0.06	0.06	0.04	0.04	0.06	0.04	-0.04	0.6384	0.02	0.04	0.04	0.06	0.02	0.04	0.03	0.05	-0.09
KETONE BODIES	Acetone	Acetone	mmol/l	0.02	0.00	0.01	0.00	0.02	0.00	0.02	0.00	0.11	0.1933	0.02	0.01	0.02	0.01	0.02	0.01	0.02	0.01	-0.02	0.8138	0.00	0.01	0.00	0.01	0.00	0.01	0.00	0.01	-0.10
LIPOPROTEIN PARTICLE CONCENTRATIONS	Total concentration of lipoprotein particles	Total-P	mmol/l	0.02	0.00	0.02	0.00	0.02	0.00	0.02	0.00	-0.03	0.7055	0.02	0.00	0.02	0.00	0.02	0.00	0.02	0.00	-0.06	0.4470	0.00	0.00	0.00	0.00	0.00	0.00	0.00	0.00	-0.10
LIPOPROTEIN PARTICLE SIZES	Average diameter for HDL particles	HDL size	nm	9.93	0.24	9.95	0.18	9.86	0.27	9.87	0.20	-0.24	**0.0069**	10.01	0.27	10.00	0.18	9.96	0.24	9.99	0.20	-0.07	0.4240	0.06	0.18	0.07	0.15	0.09	0.26	0.11	0.18	0.07
LIPOPROTEIN PARTICLE SIZES	Average diameter for LDL particles	LDL size	nm	23.96	0.08	23.97	0.07	23.95	0.08	23.96	0.06	-0.13	0.1295	23.98	0.06	23.96	0.06	23.95	0.09	23.94	0.08	-0.23	**0.0054**	-0.01	0.10	0.00	0.09	-0.03	0.11	-0.04	0.09	-0.17
LIPOPROTEIN PARTICLE SIZES	Average diameter for VLDL particles	VLDL size	nm	37.34	1.69	37.18	1.15	37.90	1.58	37.84	1.28	0.26	**0.0036**	38.32	1.17	38.36	0.96	38.55	1.79	38.61	1.31	0.12	0.1508	1.15	1.88	1.06	1.18	0.82	1.33	0.77	1.15	-0.15
LIPOPROTEIN SUBCLASSES	Cholesteryl esters in chylomicrons and extremely large VLDL	XXL-VLDL-CE	mmol/l	0.01	0.01	0.00	0.01	0.01	0.02	0.01	0.01	0.30	**0.0010**	0.05	0.04	0.05	0.03	0.05	0.04	0.06	0.03	0.14	0.0864	0.04	0.03	0.04	0.03	0.04	0.03	0.05	0.03	0.02
LIPOPROTEIN SUBCLASSES	Cholesteryl esters in IDL	IDL-CE	mmol/l	0.63	0.17	0.62	0.13	0.65	0.16	0.62	0.15	0.02	0.8485	0.99	0.26	1.02	0.22	0.94	0.28	0.97	0.23	-0.12	0.1355	0.36	0.26	0.38	0.20	0.32	0.30	0.29	0.21	-0.21
LIPOPROTEIN SUBCLASSES	Cholesteryl esters in large HDL	L-HDL-CE	mmol/l	0.42	0.18	0.45	0.12	0.38	0.20	0.39	0.14	-0.25	**0.0050**	0.46	0.21	0.47	0.16	0.43	0.20	0.44	0.17	-0.12	0.1579	0.05	0.17	0.04	0.14	0.04	0.20	0.04	0.13	-0.02
LIPOPROTEIN SUBCLASSES	Cholesteryl esters in large LDL	L-LDL-CE	mmol/l	0.80	0.25	0.80	0.17	0.85	0.24	0.81	0.20	0.14	0.1205	1.31	0.35	1.32	0.30	1.22	0.40	1.26	0.29	-0.10	0.2264	0.49	0.36	0.51	0.27	0.39	0.36	0.37	0.26	-0.25
LIPOPROTEIN SUBCLASSES	Cholesteryl esters in large VLDL	L-VLDL-CE	mmol/l	0.03	0.02	0.03	0.02	0.04	0.03	0.04	0.02	0.22	**0.0104**	0.09	0.05	0.10	0.04	0.10	0.06	0.10	0.05	0.05	0.5571	0.06	0.05	0.07	0.04	0.05	0.05	0.06	0.04	-0.07
LIPOPROTEIN SUBCLASSES	Cholesteryl esters in LDL	LDL-CE	mmol/l	1.15	0.40	1.13	0.26	1.24	0.33	1.19	0.30	0.17	0.0506	1.95	0.55	1.98	0.47	1.89	0.54	1.92	0.46	-0.07	0.3855	0.80	0.53	0.82	0.41	0.70	0.49	0.63	0.39	-0.24
LIPOPROTEIN SUBCLASSES	Concentration of chylomicrons and extremely large VLDL particles	XXL-VLDL-P	mmol/l	0.0000004	0.0000004	0.00000003	0.0000007	0.0000007	0.0000010	0.0000003	0.0000009	0.31	**0.0010**	0.000002	0.000002	0.000003	0.000002	0.000003	0.000003	0.000003	0.000002	0.19	**0.0215**	0.000002	0.000002	0.000002	0.000002	0.000002	0.000003	0.000003	0.000002	0.07
LIPOPROTEIN SUBCLASSES	Concentration of HDL particles	HDL-P	mmol/l	0.018	0.003	0.0183	0.002	0.02	0.003	0.02	0.002	-0.05	0.5399	0.02	0.004	0.02	0.003	0.02	0.004	0.02	0.003	-0.06	0.4441	0.002	0.004	0.002	0.003	0.002	0.004	0.001	0.003	-0.08
LIPOPROTEIN SUBCLASSES	Concentration of IDL particles	IDL-P	mmol/l	0.0003	0.00008	0.0003	0.00006	0.0003	0.00007	0.0003	0.00006	-0.05	0.6034	0.0005	0.0001	0.0005	0.0001	0.0005	0.0001	0.0005	0.0001	-0.08	0.3128	0.0002	0.0001	0.0002	0.0001	0.0002	0.0001	0.0002	0.0001	-0.19
LIPOPROTEIN SUBCLASSES	Concentration of large HDL particles	L-HDL-P	mmol/l	0.003	0.001	0.0028	0.001	0.002	0.001	0.002	0.001	-0.23	**0.0079**	0.003	0.001	0.003	0.001	0.003	0.001	0.003	0.001	-0.09	0.2538	0.0006	0.001	0.0006	0.0008	0.0005	0.0013	0.0006	0.0008	-0.04
LIPOPROTEIN SUBCLASSES	Concentration of large LDL particles	L-LDL-P	mmol/l	0.001	0.0002	0.0006	0.0001	0.001	0.0002	0.001	0.0002	0.13	0.1274	0.001	0.0004	0.001	0.0003	0.001	0.0004	0.001	0.0003	-0.07	0.3922	0.0005	0.0003	0.0005	0.0003	0.0004	0.0004	0.0004	0.0002	-0.20
LIPOPROTEIN SUBCLASSES	Concentration of large VLDL particles	L-VLDL-P	mmol/l	0.00001	0.00001	0.000006	0.000004	0.000009	0.000007	0.000008	0.000005	-0.26	**0.0035**	0.00002	0.00001	0.00002	0.00001	0.00002	0.00002	0.00002	0.00001	0.11	0.1919	0.000013	0.000011	0.000014	0.000009	0.000011	0.000011	0.000014	0.000010	-0.04
LIPOPROTEIN SUBCLASSES	Concentration of LDL particles	LDL-P	mmol/l	0.0010	0.0003	0.0010	0.0002	0.001	0.0003	0.001	0.0002	0.17	**0.0499**	0.002	0.001	0.002	0.0005	0.002	0.001	0.002	0.0005	-0.05	0.5201	0.0008	0.0005	0.0008	0.0004	0.0007	0.0005	0.0007	0.0004	-0.19
LIPOPROTEIN SUBCLASSES	Concentration of medium HDL particles	M-HDL-P	mmol/l	0.0052	0.001	0.0053	0.001	0.005	0.001	0.005	0.001	-0.11	0.2197	0.01	0.001	0.01	0.001	0.01	0.002	0.01	0.001	-0.07	0.4240	0.0004	0.0013	0.0004	0.0011	0.0005	0.0013	0.0003	0.0009	-0.05
LIPOPROTEIN SUBCLASSES	Concentration of medium LDL particles	M-LDL-P	mmol/l	0.0002	0.00009	0.0002	0.00006	0.0003	0.00006	0.0002	0.00006	0.22	**0.0137**	0.0004	0.0001	0.0004	0.0001	0.0004	0.0002	0.0004	0.0001	-0.02	0.8251	0.0002	0.0001	0.0002	0.0001	0.0002	0.0001	0.0002	0.0001	-0.16
LIPOPROTEIN SUBCLASSES	Concentration of medium VLDL particles	M-VLDL-P	mmol/l	0.00003	0.00001	0.00002	0.000009	0.00003	0.000014	0.00003	0.000011	0.21	**0.0192**	0.00006	0.00003	0.00006	0.00002	0.0001	0.00003	0.0001	0.00002	-0.02	0.8432	0.00004	0.00003	0.00004	0.00002	0.00003	0.00002	0.00003	0.00002	-0.13
LIPOPROTEIN SUBCLASSES	Concentration of small HDL particles	S-HDL-P	mmol/l	0.0100	0.001	0.0100	0.001	0.010	0.0015	0.010	0.0013	0.10	0.2533	0.01	0.002	0.01	0.001	0.01	0.002	0.01	0.002	0.00	0.9679	0.0006	0.0019	0.0008	0.0014	0.0002	0.0022	0.0003	0.0017	-0.13
LIPOPROTEIN SUBCLASSES	Concentration of small LDL particles	S-LDL-P	mmol/l	0.0001	0.00004	0.0001	0.00003	0.0002	0.00003	0.0001	0.00003	0.20	**0.0243**	0.0002	0.0001	0.0003	0.0001	0.0002	0.0001	0.0003	0.0001	0.00	0.9844	0.0001	0.0001	0.0001	0.0001	0.0001	0.0001	0.0001	0.00000	-0.13

				1ST TRIMESTER (N=185)										DELIVERY (N=201)										CHANGE BETWEEN DELIVERY AND 1ST TRIMESTER (DELTA) (N=150)								
				CONTROL (N=115)				SSRI (N=70)						CONTROL (N=112)				SSRI (N=89)						CONTROL (N=110)				SSRI (N=40)				
GROUP	VARIABLE	ABBRE-VIATION	UNIT	MEDIAN	IQR	MEAN	SD	MEDIAN	IQR	MEAN	SD	r	p	MEDIAN	IQR	MEAN	SD	MEDIAN	IQR	MEAN	SD	r	p	MEDIAN	IQR	MEAN	SD	MEDIAN	IQR	MEAN	SD	r
LIPOPROTEIN SUBCLASSES	Concentration of small VLDL particles	S-VLDL-P	mmol/l	0.00003	0.000 01	0.0000	0.000 01	0.00004	0.000 02	0.00004	0.000 01	0.19	**0.0291**	0.00008	0.0000 4	0.0000 9	0.0000 3	0.0001	0.000 04	0.0001	0.000 03	0.04	0.6099	0.00005	0.000 03	0.0000 5	0.000 03	0.00004	0.0000 4	0.00005	0.000 02	-0.08
LIPOPROTEIN SUBCLASSES	Concentration of very large HDL particles	XL-HDL-P	mmol/l	0.0004	0.000 2	0.0004	0.000 1	0.0003	0.000 15	0.0003	0.000 13	-0.19	**0.0322**	0.0005	0.0002	0.0005	0.0001	0.001	0.000 2	0.001	0.000 2	-0.07	0.4058	0.0002	0.000 1	0.0002	0.000 1	0.0002	0.0002	0.0002	0.000 1	0.02
LIPOPROTEIN SUBCLASSES	Concentration of very large VLDL particles	XL-VLDL-P	mmol/l	0.000002	0.000 002	0.00000 2	0.000 002	0.000003	0.000 00	0.00000 2	0.000 00	0.28	**0.0015**	0.000007	0.0000 04	0.0000 07	0.0000 04	0.000008	0.000 01	0.00001	0.000 004	0.14	0.0933	0.000005	0.000 004	0.0000 05	0.000 003	0.000004	0.0000 05	0.00000 6	0.000 004	-0.01
LIPOPROTEIN SUBCLASSES	Concentration of very small VLDL particles	XS-VLDL-P	mmol/l	0.0001	0.000 02	0.0001	0.000 01	0.0001	0.000 02	0.0001	0.000 01	0.07	0.4052	0.0001	0.0000 4	0.0001	0.0000 3	0.0001	0.000 04	0.0001	0.000 03	0.00	0.9786	0.00006	0.000 03	0.0000 6	0.000 03	0.00005	0.0000 3	0.00006	0.000 02	-0.13
LIPOPROTEIN SUBCLASSES	Concentration of VLDL particles	VLDL-P	mmol/l	0.0001	0.000 05	0.0001	0.000 04	0.0001	0.000 05	0.0001	0.000 04	0.20	**0.0259**	0.0003	0.0001	0.0003	0.0001	0.0003	0.000 1	0.0003	0.000 1	0.04	0.6254	0.0002	0.000 1	0.0002	0.000 08	0.0001	0.0001	0.0002	0.000 07	-0.09
LIPOPROTEIN SUBCLASSES	Free cholesterol in chylomicrons and extremely large VLDL	XXL-VLDL-FC	mmol/l	0.005	0.004	0.0013	0.007	0.008	0.01	0.005	0.01	0.28	**0.0012**	0.03	0.02	0.03	0.02	0.04	0.04	0.04127 8	0.000 002	0.16	0.0552	0.03	0.02	0.03	0.02	0.03	0.02	0.03	0.03	0.03
LIPOPROTEIN SUBCLASSES	Free cholesterol in IDL	IDL-FC	mmol/l	0.21	0.05	0.20	0.04	0.21	0.05	0.21	0.05	0.00	0.9898	0.33	0.09	0.34	0.07	0.32	0.11	0.32	0.08	-0.12	0.1304	0.12	0.09	0.13	0.07	0.10	0.10	0.10	0.07	-0.21
LIPOPROTEIN SUBCLASSES	Free cholesterol in large HDL	L-HDL-FC	mmol/l	0.13	0.05	0.13	0.04	0.11	0.06	0.12	0.04	-0.23	**0.0098**	0.15	0.07	0.15	0.05	0.14	0.05	0.15	0.05	-0.10	0.2408	0.03	0.05	0.03	0.04	0.02	0.06	0.03	0.04	-0.04
LIPOPROTEIN SUBCLASSES	Free cholesterol in large LDL	L-LDL-FC	mmol/l	0.29	0.09	0.28	0.06	0.30	0.06	0.29	0.07	0.08	0.3480	0.43	0.12	0.43	0.10	0.40	0.13	0.41	0.10	-0.14	0.0841	0.13	0.12	0.15	0.09	0.12	0.13	0.10	0.10	-0.23
LIPOPROTEIN SUBCLASSES	Free cholesterol in large VLDL	L-VLDL-FC	mmol/l	0.03	0.02	0.02	0.02	0.04	0.03	0.03	0.02	0.27	**0.0021**	0.08	0.05	0.09	0.04	0.09	0.07	0.10	0.05	0.10	0.2208	0.06	0.05	0.06	0.04	0.04	0.05	0.06	0.04	-0.06
LIPOPROTEIN SUBCLASSES	Free cholesterol in medium HDL	M-HDL-FC	mmol/l	0.13	0.03	0.13	0.02	0.12	0.03	0.12	0.02	-0.12	0.1764	0.15	0.04	0.14	0.03	0.14	0.04	0.14	0.03	-0.06	0.4797	0.02	0.04	0.02	0.03	0.02	0.04	0.01	0.03	-0.07
LIPOPROTEIN SUBCLASSES	Free cholesterol in medium LDL	M-LDL-FC	mmol/l	0.11	0.04	0.10	0.03	0.11	0.03	0.11	0.03	0.16	0.0747	0.16	0.05	0.17	0.05	0.16	0.05	0.16	0.04	-0.10	0.2127	0.06	0.05	0.06	0.04	0.05	0.05	0.04	0.04	-0.22
LIPOPROTEIN SUBCLASSES	Free cholesterol in medium VLDL	M-VLDL-FC	mmol/l	0.05	0.03	0.05	0.02	0.06	0.03	0.06	0.02	0.19	**0.0290**	0.14	0.07	0.14	0.05	0.14	0.07	0.14	0.05	-0.01	0.8884	0.08	0.06	0.09	0.05	0.07	0.06	0.08	0.04	-0.15
LIPOPROTEIN SUBCLASSES	Free cholesterol in small HDL	S-HDL-FC	mmol/l	0.13	0.02	0.13	0.01	0.13	0.02	0.13	0.02	0.08	0.3918	0.18	0.03	0.17	0.02	0.17	0.03	0.17	0.02	0.01	0.9484	0.04	0.03	0.04	0.02	0.04	0.03	0.04	0.02	-0.09
LIPOPROTEIN SUBCLASSES	Free cholesterol in small LDL	S-LDL-FC	mmol/l	0.04	0.02	0.04	0.01	0.05	0.01	0.05	0.01	0.13	0.1484	0.06	0.02	0.06	0.02	0.06	0.02	0.06	0.02	-0.08	0.3210	0.02	0.02	0.02	0.01	0.02	0.01	0.02	0.01	-0.17
LIPOPROTEIN SUBCLASSES	Free cholesterol in small VLDL	S-VLDL-FC	mmol/l	0.04	0.02	0.04	0.01	0.05	0.02	0.05	0.02	0.16	0.0647	0.11	0.05	0.11	0.04	0.10	0.05	0.11	0.04	-0.04	0.6454	0.06	0.04	0.07	0.03	0.05	0.05	0.06	0.03	-0.15
LIPOPROTEIN SUBCLASSES	Free cholesterol in very large HDL	XL-HDL-FC	mmol/l	0.03	0.01	0.03	0.01	0.03	0.01	0.03	0.01	-0.18	**0.0392**	0.04	0.01	0.04	0.01	0.04	0.01	0.04	0.01	-0.08	0.3023	0.01	0.01	0.01	0.01	0.01	0.01	0.01	0.01	0.03
LIPOPROTEIN SUBCLASSES	Free cholesterol in very large VLDL	XL-VLDL-FC	mmol/l	0.01	0.01	0.01	0.01	0.02	0.01	0.01	0.01	0.29	**0.0011**	0.04	0.03	0.04	0.02	0.04	0.04	0.05	0.03	0.10	0.2021	0.03	0.03	0.03	0.02	0.03	0.03	0.03	0.02	-0.04
LIPOPROTEIN SUBCLASSES	Free cholesterol in very small VLDL	XS-VLDL-FC	mmol/l	0.05	0.02	0.05	0.01	0.05	0.02	0.05	0.01	0.07	0.4239	0.12	0.04	0.12	0.03	0.12	0.04	0.12	0.03	-0.01	0.9484	0.07	0.03	0.07	0.03	0.06	0.03	0.06	0.03	-0.12
LIPOPROTEIN SUBCLASSES	Phospholipids in chylomicrons and extremely large VLDL	XXL-VLDL-PL	mmol/l	0.00	0.00	0.00	0.01	0.01	0.01	0.00	0.02	0.31	**0.0010**	0.04	0.04	0.05	0.03	0.06	0.05	0.06	0.04	0.17	**0.0383**	0.04	0.04	0.05	0.03	0.04	0.04	0.05	0.04	0.05
LIPOPROTEIN SUBCLASSES	Phospholipids in HDL	HDL-PL	mmol/l	2.04	0.41	2.09	0.28	1.97	0.42	2.02	0.32	-0.14	0.1051	2.30	0.52	2.30	0.42	2.25	0.51	2.26	0.39	-0.06	0.4997	0.27	0.49	0.26	0.39	0.23	0.47	0.21	0.32	-0.06
LIPOPROTEIN SUBCLASSES	Phospholipids in IDL	IDL-PL	mmol/l	0.28	0.08	0.28	0.06	0.29	0.07	0.28	0.06	0.04	0.6763	0.47	0.12	0.47	0.10	0.44	0.15	0.45	0.11	-0.10	0.2302	0.18	0.11	0.19	0.09	0.13	0.12	0.15	0.10	-0.23
LIPOPROTEIN SUBCLASSES	Phospholipids in large HDL	L-HDL-PL	mmol/l	0.57	0.22	0.61	0.14	0.52	0.20	0.54	0.16	-0.23	**0.0092**	0.65	0.23	0.65	0.19	0.62	0.21	0.62	0.19	-0.09	0.2768	0.09	0.22	0.08	0.16	0.08	0.23	0.07	0.15	-0.03
LIPOPROTEIN SUBCLASSES	Phospholipids in large LDL	L-LDL-PL	mmol/l	0.33	0.10	0.32	0.07	0.34	0.07	0.33	0.07	0.12	0.1667	0.51	0.13	0.52	0.12	0.49	0.14	0.50	0.12	-0.09	0.2833	0.19	0.13	0.19	0.10	0.16	0.13	0.15	0.10	-0.21
LIPOPROTEIN SUBCLASSES	Phospholipids in large VLDL	L-VLDL-PL	mmol/l	0.04	0.04	0.03	0.03	0.06	0.05	0.05	0.04	0.25	**0.0044**	0.13	0.08	0.14	0.07	0.14	0.11	0.16	0.08	0.12	0.1543	0.10	0.08	0.10	0.06	0.08	0.08	0.10	0.07	-0.03
LIPOPROTEIN SUBCLASSES	Phospholipids in LDL	LDL-PL	mmol/l	0.55	0.18	0.53	0.11	0.58	0.14	0.57	0.12	0.15	0.0839	0.88	0.25	0.90	0.21	0.85	0.23	0.88	0.21	-0.07	0.4142	0.34	0.24	0.36	0.18	0.31	0.19	0.28	0.18	-0.20
LIPOPROTEIN SUBCLASSES	Phospholipids in medium HDL	M-HDL-PL	mmol/l	0.62	0.11	0.61	0.08	0.61	0.13	0.60	0.09	-0.05	0.5399	0.67	0.17	0.67	0.13	0.66	0.14	0.66	0.11	-0.02	0.7855	0.05	0.14	0.05	0.12	0.03	0.14	0.03	0.10	-0.06
LIPOPROTEIN SUBCLASSES	Phospholipids in medium LDL	M-LDL-PL	mmol/l	0.14	0.05	0.14	0.03	0.15	0.04	0.15	0.04	0.19	**0.0269**	0.25	0.08	0.25	0.07	0.24	0.08	0.25	0.06	-0.03	0.6952	0.10	0.07	0.11	0.06	0.09	0.07	0.09	0.05	-0.17
LIPOPROTEIN SUBCLASSES	Phospholipids in medium VLDL	M-VLDL-PL	mmol/l	0.09	0.05	0.09	0.04	0.11	0.05	0.10	0.04	0.20	**0.0226**	0.24	0.12	0.24	0.08	0.24	0.11	0.25	0.09	0.01	0.8961	0.14	0.10	0.15	0.08	0.13	0.09	0.13	0.07	-0.14
LIPOPROTEIN SUBCLASSES	Phospholipids in small HDL	S-HDL-PL	mmol/l	0.71	0.09	0.71	0.07	0.72	0.12	0.71	0.10	0.09	0.3238	0.78	0.15	0.80	0.11	0.80	0.12	0.80	0.11	0.05	0.5425	0.08	0.14	0.09	0.11	0.07	0.13	0.06	0.10	-0.10
LIPOPROTEIN SUBCLASSES	Phospholipids in small LDL	S-LDL-PL	mmol/l	0.08	0.02	0.08	0.02	0.08	0.02	0.08	0.02	0.14	0.1185	0.13	0.04	0.13	0.03	0.13	0.04	0.13	0.03	-0.01	0.9290	0.05	0.04	0.05	0.03	0.04	0.03	0.05	0.02	-0.14
LIPOPROTEIN SUBCLASSES	Phospholipids in small VLDL	S-VLDL-PL	mmol/l	0.08	0.03	0.07	0.02	0.09	0.03	0.08	0.03	0.18	**0.0437**	0.19	0.08	0.19	0.06	0.19	0.09	0.19	0.06	0.00	0.9659	0.11	0.07	0.11	0.05	0.10	0.08	0.10	0.05	-0.11
LIPOPROTEIN SUBCLASSES	Phospholipids in very large HDL	XL-HDL-PL	mmol/l	0.14	0.07	0.14	0.05	0.12	0.06	0.12	0.06	-0.22	**0.0109**	0.18	0.08	0.18	0.06	0.17	0.07	0.17	0.06	-0.10	0.2172	0.04	0.06	0.04	0.05	0.04	0.07	0.05	0.05	0.02
LIPOPROTEIN SUBCLASSES	Phospholipids in very large VLDL	XL-VLDL-PL	mmol/l	0.02	0.02	0.01	0.02	0.03	0.02	0.02	0.02	0.29	**0.0011**	0.07	0.05	0.07	0.04	0.08	0.07	0.09	0.05	0.12	0.1368	0.06	0.05	0.06	0.04	0.04	0.05	0.06	0.04	-0.02
LIPOPROTEIN SUBCLASSES	Phospholipids in very small VLDL	XS-VLDL-PL	mmol/l	0.10	0.03	0.10	0.03	0.10	0.04	0.10	0.03	0.08	0.3871	0.23	0.08	0.23	0.06	0.23	0.09	0.24	0.07	0.00	0.9581	0.13	0.07	0.14	0.05	0.12	0.06	0.13	0.05	-0.09
LIPOPROTEIN SUBCLASSES	Phospholipids in VLDL	VLDL-PL	mmol/l	0.33	0.17	0.31	0.13	0.39	0.20	0.36	0.15	0.22	**0.0112**	0.91	0.44	0.93	0.32	0.95	0.51	0.98	0.35	0.07	0.4017	0.58	0.41	0.61	0.29	0.49	0.40	0.58	0.28	-0.06
LIPOPROTEIN SUBCLASSES	Total lipids in chylomicrons and extremely large VLDL	XXL-VLDL-L	mmol/l	0.04	0.03	0.01	0.09	0.08	0.11	0.02	0.12	0.28	**0.0013**	0.26	0.25	0.31	0.24	0.34	0.40	0.41	0.31	0.18	**0.0272**	0.22	0.25	0.27	0.23	0.24	0.34	0.33	0.32	0.06
LIPOPROTEIN SUBCLASSES	Total lipids in IDL	IDL-L	mmol/l	1.24	0.32	1.21	0.25	1.27	0.30	1.20	0.28	0.02	0.8000	2.03	0.58	2.09	0.43	1.99	0.59	2.01	0.46	-0.10	0.2255	0.78	0.51	0.85	0.39	0.64	0.59	0.68	0.40	-0.21
LIPOPROTEIN SUBCLASSES	Total lipids in large HDL	L-HDL-L	mmol/l	1.17	0.47	1.24	0.32	1.06	0.47	1.09	0.36	-0.23	**0.0077**	1.36	0.54	1.38	0.42	1.28	0.42	1.31	0.43	-0.09	0.2599	0.21	0.46	0.20	0.35	0.18	0.51	0.20	0.32	-0.03
LIPOPROTEIN SUBCLASSES	Total lipids in large LDL	L-LDL-L	mmol/l	1.53	0.47	1.52	0.31	1.61	0.42	1.54	0.35	0.12	0.1783	2.49	0.61	2.52	0.55	2.34	0.69	2.44	0.55	-0.09	0.2661	0.96	0.61	0.99	0.49	0.83	0.64	0.76	0.49	-0.23
LIPOPROTEIN SUBCLASSES	Total lipids in large VLDL	L-VLDL-L	mmol/l	0.22	0.17	0.19	0.15	0.29	0.24	0.26	0.18	0.27	**0.0022**	0.57	0.39	0.62	0.32	0.65	0.55	0.70	0.40	0.10	0.2056	0.36	0.36	0.41	0.30	0.31	0.39	0.41	0.34	-0.05
LIPOPROTEIN SUBCLASSES	Total lipids in medium HDL	M-HDL-L	mmol/l	1.36	0.25	1.36	0.18	1.32	0.29	1.32	0.21	-0.09	0.3252	1.45	0.36	1.45	0.30	1.42	0.37	1.43	0.26	-0.05	0.5344	0.09	0.34	0.10	0.28	0.09	0.31	0.06	0.22	-0.06
LIPOPROTEIN SUBCLASSES	Total lipids in medium LDL	M-LDL-L	mmol/l	0.53	0.20	0.50	0.14	0.58	0.16	0.56	0.15	0.21	**0.0144**	0.96	0.28	0.97	0.26	0.92	0.33	0.96	0.26	-0.02	0.7724	0.43	0.30	0.45	0.22	0.39	0.27	0.37	0.21	-0.18
LIPOPROTEIN SUBCLASSES	Total lipids in medium VLDL	M-VLDL-L	mmol/l	0.44	0.21	0.40	0.17	0.51	0.23	0.50	0.20	0.23	**0.0095**	1.02	0.47	1.06	0.38	1.02	0.61	1.10	0.43	0.03	0.7079	0.62	0.48	0.63	0.34	0.50	0.55	0.57	0.34	-0.11

				1ST TRIMESTER (N=185)										DELIVERY (N=201)										CHANGE BETWEEN DELIVERY AND 1ST TRIMESTER (DELTA) (N=150)								
				CONTROL (N=115)				SSRI (N=70)						CONTROL (N=112)				SSRI (N=89)						CONTROL (N=110)				SSRI (N=40)				
GROUP	VARIABLE	ABBRE-VIATION	UNIT	MEDIAN	IQR	MEAN	SD	MEDIAN	IQR	MEAN	SD	r	p	MEDIAN	IQR	MEAN	SD	MEDIAN	IQR	MEAN	SD	r	p	MEDIAN	IQR	MEAN	SD	MEDIAN	IQR	MEAN	SD	r
LIPOPROTEIN SUBCLASSES	Total lipids in small HDL	S-HDL-L	mmol/l	1.22	0.16	1.22	0.12	1.25	0.21	1.23	0.16	0.09	0.2982	1.36	0.25	1.39	0.18	1.39	0.23	1.40	0.18	0.05	0.5248	0.14	0.24	0.17	0.17	0.13	0.23	0.12	0.18	-0.10
LIPOPROTEIN SUBCLASSES	Total lipids in small LDL	S-LDL-L	mmol/l	0.24	0.08	0.23	0.05	0.26	0.06	0.26	0.06	0.18	**0.0381**	0.41	0.13	0.43	0.11	0.41	0.14	0.42	0.10	-0.01	0.8653	0.17	0.12	0.18	0.09	0.16	0.10	0.16	0.08	-0.15
LIPOPROTEIN SUBCLASSES	Total lipids in small VLDL	S-VLDL-L	mmol/l	0.35	0.15	0.33	0.11	0.39	0.15	0.38	0.12	0.19	**0.0300**	0.85	0.35	0.88	0.27	0.86	0.39	0.90	0.30	0.04	0.6630	0.50	0.32	0.53	0.25	0.44	0.36	0.50	0.24	-0.08
LIPOPROTEIN SUBCLASSES	Total lipids in very large HDL	XL-HDL-L	mmol/l	0.27	0.13	0.27	0.09	0.24	0.12	0.24	0.10	-0.21	**0.0147**	0.36	0.15	0.36	0.10	0.33	0.13	0.34	0.12	-0.10	0.2369	0.08	0.11	0.09	0.09	0.09	0.13	0.09	0.09	0.01
LIPOPROTEIN SUBCLASSES	Total lipids in very large VLDL	XL-VLDL-L	mmol/l	0.10	0.11	0.08	0.10	0.15	0.14	0.13	0.12	0.29	**0.0011**	0.35	0.27	0.38	0.21	0.39	0.34	0.45	0.26	0.14	0.0815	0.26	0.21	0.28	0.20	0.23	0.28	0.29	0.23	-0.01
LIPOPROTEIN SUBCLASSES	Total lipids in very small VLDL	XS-VLDL-L	mmol/l	0.34	0.10	0.33	0.09	0.36	0.13	0.34	0.09	0.07	0.4141	0.76	0.23	0.76	0.19	0.73	0.27	0.77	0.20	0.00	0.9893	0.41	0.20	0.42	0.17	0.36	0.20	0.39	0.16	-0.12
LIPOPROTEIN SUBCLASSES	Total lipids in VLDL	VLDL-L	mmol/l	1.49	0.73	1.35	0.63	1.78	0.93	1.67	0.75	0.25	**0.0050**	3.96	1.91	4.01	1.47	3.97	2.40	4.33	1.71	0.09	0.2682	2.40	1.80	2.54	1.34	2.05	2.00	2.49	1.40	-0.04
LIPOPROTEIN SUBCLASSES	Total phospholipids in lipoprotein particles	Total-PL	mmol/l	3.19	0.55	3.18	0.40	3.22	0.69	3.16	0.49	0.03	0.7717	4.64	0.86	4.61	0.65	4.51	0.99	4.58	0.69	-0.05	0.5216	1.37	0.85	1.42	0.63	1.16	0.99	1.22	0.60	-0.16
LIPOPROTEIN SUBCLASSES	Triglycerides in chylomicrons and extremely large VLDL	XXL-VLDL-TG	mmol/l	0.03	0.02	0.00	0.06	0.05	0.07	0.01	0.08	0.30	**0.0010**	0.13	0.18	0.17	0.17	0.19	0.28	0.24	0.22	0.18	**0.0247**	0.12	0.16	0.15	0.16	0.14	0.26	0.20	0.22	0.08
LIPOPROTEIN SUBCLASSES	Triglycerides in IDL	IDL-TG	mmol/l	0.12	0.03	0.11	0.03	0.12	0.03	0.12	0.03	0.10	0.2395	0.25	0.08	0.26	0.06	0.26	0.09	0.27	0.07	0.07	0.4045	0.14	0.07	0.14	0.06	0.13	0.07	0.14	0.06	-0.01
LIPOPROTEIN SUBCLASSES	Triglycerides in large HDL	L-HDL-TG	mmol/l	0.05	0.02	0.05	0.02	0.05	0.02	0.05	0.02	0.02	0.8308	0.10	0.04	0.10	0.03	0.10	0.03	0.11	0.03	0.05	0.5621	0.05	0.03	0.05	0.02	0.05	0.03	0.05	0.02	-0.01
LIPOPROTEIN SUBCLASSES	Triglycerides in large VLDL	L-VLDL-TG	mmol/l	0.12	0.10	0.10	0.09	0.16	0.13	0.14	0.10	0.27	**0.0019**	0.26	0.22	0.30	0.18	0.30	0.30	0.34	0.23	0.10	0.2056	0.15	0.19	0.18	0.17	0.14	0.22	0.19	0.20	-0.03
LIPOPROTEIN SUBCLASSES	Triglycerides in LDL	LDL-TG	mmol/l	0.16	0.04	0.16	0.04	0.17	0.05	0.17	0.04	0.12	0.1720	0.37	0.12	0.38	0.09	0.37	0.14	0.39	0.10	0.09	0.2978	0.21	0.10	0.21	0.08	0.20	0.10	0.21	0.09	0.00
LIPOPROTEIN SUBCLASSES	Triglycerides in medium HDL	M-HDL-TG	mmol/l	0.06	0.02	0.06	0.02	0.07	0.03	0.07	0.02	0.15	0.0844	0.13	0.04	0.13	0.03	0.13	0.04	0.13	0.03	0.11	0.1727	0.07	0.04	0.06	0.03	0.06	0.03	0.06	0.03	-0.05
LIPOPROTEIN SUBCLASSES	Triglycerides in medium LDL	M-LDL-TG	mmol/l	0.04	0.01	0.04	0.01	0.04	0.01	0.04	0.01	0.15	0.0951	0.09	0.03	0.09	0.02	0.08	0.04	0.09	0.03	0.09	0.2746	0.05	0.03	0.05	0.02	0.05	0.03	0.05	0.02	0.02
LIPOPROTEIN SUBCLASSES	Triglycerides in medium VLDL	M-VLDL-TG	mmol/l	0.23	0.14	0.21	0.11	0.28	0.16	0.26	0.12	0.24	**0.0066**	0.50	0.28	0.54	0.22	0.55	0.36	0.58	0.28	0.09	0.2630	0.29	0.26	0.31	0.21	0.24	0.29	0.30	0.23	-0.05
LIPOPROTEIN SUBCLASSES	Triglycerides in small HDL	S-HDL-TG	mmol/l	0.05	0.02	0.05	0.02	0.06	0.02	0.05	0.02	0.21	**0.0153**	0.11	0.04	0.11	0.03	0.11	0.04	0.12	0.03	0.14	0.0828	0.06	0.04	0.06	0.03	0.06	0.04	0.06	0.03	0.00
LIPOPROTEIN SUBCLASSES	Triglycerides in small LDL	S-LDL-TG	mmol/l	0.01	0.00	0.01	0.00	0.02	0.01	0.02	0.00	0.21	**0.0166**	0.04	0.01	0.04	0.01	0.04	0.01	0.04	0.01	0.14	0.1006	0.02	0.01	0.02	0.01	0.02	0.01	0.02	0.01	0.05
LIPOPROTEIN SUBCLASSES	Triglycerides in small VLDL	S-VLDL-TG	mmol/l	0.16	0.07	0.15	0.06	0.18	0.07	0.17	0.06	0.18	**0.0403**	0.37	0.17	0.38	0.12	0.40	0.20	0.41	0.15	0.10	0.2418	0.22	0.15	0.22	0.11	0.21	0.16	0.23	0.13	-0.01
LIPOPROTEIN SUBCLASSES	Triglycerides in very large HDL	XL-HDL-TG	mmol/l	0.01	0.00	0.01	0.00	0.01	0.00	0.01	0.00	0.03	0.6992	0.02	0.01	0.02	0.01	0.02	0.01	0.02	0.01	0.09	0.3001	0.01	0.01	0.01	0.01	0.01	0.01	0.01	0.01	0.05
LIPOPROTEIN SUBCLASSES	Triglycerides in very large VLDL	XL-VLDL-TG	mmol/l	0.06	0.07	0.04	0.06	0.09	0.09	0.07	0.07	0.29	**0.0010**	0.19	0.15	0.21	0.13	0.23	0.21	0.26	0.17	0.16	0.0510	0.13	0.12	0.15	0.12	0.14	0.20	0.17	0.15	0.01
LIPOPROTEIN SUBCLASSES	Triglycerides in very small VLDL	XS-VLDL-TG	mmol/l	0.08	0.02	0.07	0.02	0.08	0.03	0.08	0.02	0.13	0.1406	0.18	0.06	0.19	0.05	0.19	0.09	0.20	0.06	0.08	0.3568	0.11	0.06	0.11	0.05	0.10	0.07	0.11	0.05	0.00
LIPOPROTEIN SUBCLASSES (LARGE HDL)	Cholesterol in large HDL	L-HDL-C	mmol/l	0.55	0.23	0.59	0.16	0.49	0.26	0.50	0.19	-0.24	**0.0054**	0.61	0.28	0.62	0.21	0.57	0.25	0.58	0.22	-0.11	0.1705	0.07	0.23	0.07	0.17	0.06	0.27	0.07	0.16	-0.03
LIPOPROTEIN SUBCLASSES (CHYLOMICRONS AND EXTREMELY LARGE LDL)	Cholesterol in chylomicrons and extremely large VLDL	XXL-VLDL-C	mmol/l	0.01	0.01	0.01	0.02	0.02	0.03	0.01	0.03	0.29	**0.0010**	0.08	0.06	0.09	0.05	0.09	0.07	0.10	0.06	0.15	0.0749	0.07	0.05	0.07	0.04	0.06	0.06	0.08	0.05	0.03
LIPOPROTEIN SUBCLASSES (IDL)	Cholesterol in IDL	IDL-C	mmol/l	0.84	0.23	0.82	0.18	0.86	0.20	0.82	0.20	0.01	0.8908	1.31	0.36	1.36	0.29	1.27	0.39	1.29	0.31	-0.12	0.1311	0.48	0.35	0.51	0.27	0.41	0.40	0.39	0.28	-0.21
LIPOPROTEIN SUBCLASSES (LARGE LDL)	Cholesterol in large LDL	L-LDL-C	mmol/l	1.09	0.35	1.08	0.23	1.15	0.30	1.10	0.26	0.12	0.1702	1.72	0.46	1.75	0.39	1.63	0.48	1.67	0.39	-0.11	0.1735	0.63	0.47	0.66	0.35	0.50	0.50	0.47	0.36	-0.25
LIPOPROTEIN SUBCLASSES (LARGE VLDL)	Cholesterol in large VLDL	L-VLDL-C	mmol/l	0.06	0.05	0.05	0.04	0.07	0.06	0.07	0.04	0.25	**0.0050**	0.17	0.10	0.18	0.08	0.19	0.13	0.20	0.10	0.08	0.3593	0.12	0.10	0.13	0.08	0.09	0.10	0.12	0.08	-0.06
LIPOPROTEIN SUBCLASSES (MEDIUM HDL)	Cholesterol in medium HDL	M-HDL-C	mmol/l	0.67	0.14	0.68	0.09	0.64	0.14	0.65	0.11	-0.15	0.0814	0.67	0.18	0.66	0.16	0.61	0.24	0.63	0.15	-0.10	0.2190	-0.03	0.17	-0.01	0.15	-0.02	0.18	-0.03	0.12	-0.06
LIPOPROTEIN SUBCLASSES (MEDIUM HDL)	Cholesteryl esters in medium HDL	M-HDL-CE	mmol/l	0.55	0.11	0.55	0.07	0.52	0.11	0.53	0.08	-0.16	0.0623	0.53	0.15	0.51	0.13	0.48	0.19	0.49	0.12	-0.10	0.2021	-0.04	0.14	-0.03	0.12	-0.03	0.13	-0.05	0.10	-0.06
LIPOPROTEIN SUBCLASSES (MEDIUM HDL)	Cholesteryl esters in medium LDL	M-LDL-CE	mmol/l	0.24	0.10	0.23	0.07	0.27	0.08	0.27	0.08	0.24	**0.0064**	0.47	0.16	0.47	0.13	0.46	0.17	0.47	0.13	-0.01	0.8730	0.21	0.15	0.23	0.11	0.19	0.14	0.19	0.11	-0.18
LIPOPROTEIN SUBCLASSES (MEDIUM HDL)	Cholesteryl esters in medium VLDL	M-VLDL-CE	mmol/l	0.06	0.03	0.06	0.03	0.06	0.03	0.06	0.03	0.07	0.4109	0.13	0.07	0.14	0.06	0.12	0.07	0.13	0.05	-0.12	0.1460	0.07	0.06	0.08	0.05	0.05	0.06	0.06	0.04	-0.20
LIPOPROTEIN SUBCLASSES (MEDIUM HDL)	Cholesteryl esters in small HDL	S-HDL-CE	mmol/l	0.33	0.05	0.33	0.04	0.34	0.06	0.33	0.05	0.06	0.4626	0.30	0.08	0.30	0.06	0.30	0.08	0.30	0.06	-0.05	0.5720	-0.04	0.07	-0.03	0.05	-0.05	0.08	-0.04	0.06	-0.16
LIPOPROTEIN SUBCLASSES (MEDIUM HDL)	Cholesteryl esters in small LDL	S-LDL-CE	mmol/l	0.11	0.03	0.10	0.03	0.12	0.03	0.11	0.03	0.21	**0.0171**	0.19	0.06	0.19	0.05	0.18	0.07	0.19	0.05	-0.02	0.8100	0.08	0.05	0.09	0.04	0.08	0.05	0.07	0.04	-0.17
LIPOPROTEIN SUBCLASSES (MEDIUM HDL)	Cholesteryl esters in small VLDL	S-VLDL-CE	mmol/l	0.07	0.03	0.06	0.02	0.08	0.03	0.07	0.03	0.17	**0.0499**	0.19	0.08	0.19	0.07	0.19	0.09	0.19	0.07	-0.01	0.8787	0.12	0.07	0.13	0.06	0.11	0.09	0.11	0.05	-0.12
LIPOPROTEIN SUBCLASSES (MEDIUM HDL)	Cholesteryl esters in very large HDL	XL-HDL-CE	mmol/l	0.09	0.04	0.09	0.03	0.08	0.04	0.08	0.03	-0.22	**0.0129**	0.12	0.05	0.12	0.03	0.11	0.04	0.11	0.04	-0.12	0.1361	0.02	0.03	0.02	0.03	0.02	0.04	0.02	0.03	-0.01
LIPOPROTEIN SUBCLASSES (MEDIUM HDL)	Cholesteryl esters in very large VLDL	XL-VLDL-CE	mmol/l	0.01	0.01	0.01	0.01	0.02	0.01	0.02	0.01	0.25	**0.0048**	0.05	0.03	0.05	0.02	0.05	0.04	0.06	0.03	0.06	0.4441	0.04	0.03	0.04	0.02	0.03	0.03	0.03	0.02	-0.08
LIPOPROTEIN SUBCLASSES (MEDIUM HDL)	Cholesteryl esters in very small VLDL	XS-VLDL-CE	mmol/l	0.11	0.03	0.11	0.03	0.12	0.04	0.11	0.03	0.04	0.6887	0.22	0.06	0.22	0.05	0.21	0.07	0.21	0.06	-0.07	0.3762	0.10	0.06	0.11	0.05	0.08	0.07	0.09	0.05	-0.18
LIPOPROTEIN SUBCLASSES (MEDIUM HDL)	Cholesteryl esters in VLDL	VLDL-CE	mmol/l	0.29	0.14	0.28	0.10	0.33	0.13	0.31	0.11	0.17	0.0540	0.74	0.31	0.75	0.24	0.71	0.30	0.75	0.24	-0.01	0.8576	0.43	0.28	0.46	0.22	0.38	0.31	0.40	0.18	-0.13
LIPOPROTEIN SUBCLASSES (MEDIUM LDL)	Cholesterol in medium LDL	M-LDL-C	mmol/l	0.35	0.14	0.33	0.10	0.39	0.12	0.37	0.10	0.23	**0.0100**	0.62	0.19	0.64	0.18	0.62	0.20	0.62	0.17	-0.03	0.6934	0.27	0.19	0.29	0.15	0.24	0.20	0.23	0.14	-0.19

				1ST TRIMESTER (N=185)										DELIVERY (N=201)										CHANGE BETWEEN DELIVERY AND 1ST TRIMESTER (DELTA) (N=150)								
				CONTROL (N=115)				SSRI (N=70)						CONTROL (N=112)				SSRI (N=89)						CONTROL (N=110)				SSRI (N=40)				
GROUP	VARIABLE	ABBRE-VIATION	UNIT	MEDIAN	IQR	MEAN	SD	MEDIAN	IQR	MEAN	SD	r	p	MEDIAN	IQR	MEAN	SD	MEDIAN	IQR	MEAN	SD	r	p	MEDIAN	IQR	MEAN	SD	MEDIAN	IQR	MEAN	SD	r
LIPOPROTEIN SUBCLASSES (MEDIUM VLDL)	Cholesterol in medium VLDL	M-VLDL-C	mmol/l	0.11	0.07	0.11	0.05	0.13	0.05	0.12	0.05	0.14	0.1198	0.28	0.13	0.28	0.10	0.25	0.14	0.27	0.10	-0.08	0.3619	0.16	0.11	0.17	0.09	0.13	0.12	0.14	0.08	-0.20
LIPOPROTEIN SUBCLASSES (SMALL HDL)	Cholesterol in small HDL	S-HDL-C	mmol/l	0.46	0.06	0.46	0.05	0.47	0.07	0.47	0.06	0.07	0.4044	0.48	0.08	0.48	0.07	0.47	0.10	0.47	0.08	-0.02	0.8119	0.00	0.09	0.01	0.06	-0.01	0.10	-0.01	0.08	-0.13
LIPOPROTEIN SUBCLASSES (SMALL LDL)	Cholesterol in small LDL	S-LDL-C	mmol/l	0.15	0.05	0.14	0.04	0.16	0.04	0.16	0.04	0.19	**0.0320**	0.25	0.08	0.26	0.07	0.24	0.08	0.25	0.07	-0.04	0.6401	0.10	0.07	0.11	0.06	0.09	0.06	0.09	0.05	-0.18
LIPOPROTEIN SUBCLASSES (SMALL VLDL)	Cholesterol in small VLDL	S-VLDL-C	mmol/l	0.11	0.05	0.10	0.04	0.13	0.05	0.12	0.04	0.17	0.0548	0.30	0.13	0.30	0.10	0.29	0.15	0.30	0.10	-0.02	0.7855	0.18	0.11	0.19	0.09	0.16	0.13	0.17	0.08	-0.13
LIPOPROTEIN SUBCLASSES (VERY LARGE HDL)	Cholesterol in very large HDL	XL-HDL-C	mmol/l	0.12	0.06	0.12	0.04	0.11	0.05	0.11	0.04	-0.22	**0.0135**	0.16	0.06	0.16	0.04	0.15	0.05	0.15	0.05	-0.11	0.1846	0.03	0.04	0.03	0.04	0.03	0.06	0.03	0.04	0.01
LIPOPROTEIN SUBCLASSES (VERY LARGE VLDL)	Cholesterol in very large VLDL	XL-VLDL-C	mmol/l	0.02	0.02	0.02	0.02	0.04	0.03	0.03	0.02	0.27	**0.0024**	0.09	0.05	0.10	0.04	0.10	0.07	0.10	0.05	0.08	0.3023	0.07	0.05	0.07	0.04	0.05	0.06	0.07	0.04	-0.06
LIPOPROTEIN SUBCLASSES (VERY SMALL VLDL)	Cholesterol in very small VLDL	XS-VLDL-C	mmol/l	0.17	0.05	0.17	0.04	0.17	0.05	0.16	0.04	0.05	0.5761	0.34	0.10	0.34	0.08	0.33	0.12	0.33	0.09	-0.05	0.5441	0.17	0.09	0.17	0.08	0.14	0.10	0.15	0.07	-0.16
OTHER LIPIDS	Phosphatidylcholines	Phosphatidylc	mmol/l	2.68	0.51	2.64	0.38	2.69	0.62	2.68	0.43	0.04	0.6804	3.85	0.70	3.84	0.55	3.78	0.80	3.84	0.59	-0.02	0.7817	1.15	0.67	1.17	0.54	0.95	0.85	1.02	0.51	-0.16
OTHER LIPIDS	Phosphoglycerides	Phosphoglyc	mmol/l	2.72	0.49	2.68	0.38	2.75	0.63	2.73	0.44	0.05	0.5956	3.90	0.69	3.88	0.55	3.81	0.87	3.89	0.59	-0.01	0.8807	1.12	0.69	1.16	0.55	0.91	0.86	1.01	0.51	-0.15
OTHER LIPIDS	Ratio of triglycerides to phosphoglycerides	TG/PG	%	0.41	0.15	0.38	0.13	0.47	0.19	0.44	0.15	0.25	**0.0045**	0.68	0.26	0.71	0.22	0.75	0.36	0.79	0.27	0.18	**0.0251**	0.28	0.22	0.31	0.20	0.30	0.36	0.33	0.24	0.03
OTHER LIPIDS	Sphingomyelins	Sphingomyelins	mmol/l	0.50	0.08	0.49	0.06	0.50	0.07	0.49	0.07	-0.02	0.8175	0.68	0.12	0.68	0.09	0.66	0.12	0.66	0.10	-0.08	0.3392	0.17	0.12	0.18	0.09	0.14	0.15	0.15	0.09	-0.17
OTHER LIPIDS	Total cholines	Cholines	mmol/l	3.00	0.52	3.00	0.37	3.02	0.58	3.00	0.44	0.03	0.7501	4.15	0.70	4.16	0.56	4.09	0.87	4.14	0.60	-0.04	0.6612	1.12	0.64	1.16	0.55	0.90	0.87	0.99	0.53	-0.15
RELATIVE LIPOPROTEIN LIPID CONCENTRATIONS	Free cholesterol to total lipids ratio in chylomicrons and extremely large VLDL* 5	XXL-VLDL-FC %	%											11.98	5.79	13.46	4.63	10.65	3.91	12.21	4.53	-0.20	**0.0172**									
RELATIVE LIPOPROTEIN LIPID CONCENTRATIONS	Free cholesterol to total lipids ratio in IDL	IDL-FC %	%	17.02	0.70	17.05	0.64	16.83	1.18	16.89	0.77	-0.13	0.1460	16.33	0.65	16.23	0.56	16.03	0.71	15.94	0.87	-0.23	**0.0046**	-0.77	0.83	-0.80	0.62	-0.88	0.68	-0.99	0.98	-0.14
RELATIVE LIPOPROTEIN LIPID CONCENTRATIONS	Free cholesterol to total lipids ratio in large HDL	L-HDL-FC %	%	10.55	0.52	10.56	0.44	10.41	0.68	10.47	0.65	-0.12	0.1632	11.08	0.46	11.12	0.44	11.13	0.58	11.03	0.52	-0.03	0.7079	0.51	0.57	0.56	0.46	0.52	0.65	0.52	0.60	-0.01
RELATIVE LIPOPROTEIN LIPID CONCENTRATIONS	Free cholesterol to total lipids ratio in large LDL	L-LDL-FC %	%	18.81	1.19	18.86	0.83	18.53	1.05	18.53	0.87	-0.20	**0.0264**	17.34	1.22	17.20	1.06	16.75	1.50	16.72	1.45	-0.23	**0.0045**	-1.61	1.15	-1.62	0.95	-1.84	1.67	-1.92	1.37	-0.12
RELATIVE LIPOPROTEIN LIPID CONCENTRATIONS	Free cholesterol to total lipids ratio in large VLDL	L-VLDL-FC %	%	12.24	1.97	12.32	2.12	12.43	1.27	12.60	1.37	0.08	0.3825	14.42	1.98	14.68	1.74	13.95	2.30	14.71	2.43	-0.08	0.3270	2.02	2.59	2.40	2.57	1.42	3.16	2.33	2.88	-0.09
RELATIVE LIPOPROTEIN LIPID CONCENTRATIONS	Free cholesterol to total lipids ratio in medium HDL	M-HDL-FC %	%	9.25	0.58	9.22	0.41	9.15	0.65	9.18	0.52	-0.12	0.1702	9.93	0.54	9.94	0.45	9.81	0.51	9.83	0.47	-0.15	0.0718	0.70	0.54	0.69	0.38	0.51	0.63	0.61	0.48	-0.17
RELATIVE LIPOPROTEIN LIPID CONCENTRATIONS	Free cholesterol to total lipids ratio in medium LDL	M-LDL-FC %	%	20.15	1.85	20.37	1.49	19.68	1.86	19.95	1.49	-0.19	**0.0279**	17.20	2.02	17.04	1.68	16.30	2.44	16.38	1.98	-0.23	**0.0046**	-3.07	2.19	-3.16	1.61	-3.50	2.36	-3.39	1.83	-0.07
RELATIVE LIPOPROTEIN LIPID CONCENTRATIONS	Free cholesterol to total lipids ratio in medium VLDL	M-VLDL-FC %	%	12.50	2.41	12.52	1.85	12.39	2.17	12.13	1.43	-0.06	0.4626	13.61	1.80	13.64	1.41	13.29	2.42	13.38	1.93	-0.14	0.0972	0.97	2.55	1.08	1.82	0.58	2.40	0.89	1.94	-0.08
RELATIVE LIPOPROTEIN LIPID CONCENTRATIONS	Free cholesterol to total lipids ratio in small HDL	S-HDL-FC %	%	10.81	0.86	10.69	0.66	10.74	0.77	10.66	0.70	-0.07	0.4421	12.51	1.38	12.63	1.03	12.44	1.30	12.57	1.04	-0.03	0.6755	1.62	1.10	1.83	0.87	1.76	0.98	1.77	0.89	0.01
RELATIVE LIPOPROTEIN LIPID CONCENTRATIONS	Free cholesterol to total lipids ratio in small LDL	S-LDL-FC %	%	17.89	1.54	18.15	1.74	17.71	1.62	17.87	1.43	-0.13	0.1429	15.31	1.69	15.14	1.59	14.75	2.15	14.67	1.86	-0.17	**0.0340**	-2.81	1.96	-2.79	1.58	-2.91	1.81	-2.98	1.66	-0.06
RELATIVE LIPOPROTEIN LIPID CONCENTRATIONS	Free cholesterol to total lipids ratio in small VLDL	S-VLDL-FC %	%	12.73	2.67	12.63	1.86	12.70	2.05	12.23	1.71	-0.04	0.6233	12.53	1.86	12.51	1.39	12.11	1.73	12.03	1.50	-0.22	**0.0065**	-0.26	2.08	-0.27	1.67	-0.62	2.29	-0.65	1.61	-0.11
RELATIVE LIPOPROTEIN LIPID CONCENTRATIONS	Free cholesterol to total lipids ratio in very large HDL	XL-HDL-FC %	%	11.41	1.18	11.01	1.57	11.90	1.81	11.43	1.78	0.20	**0.0219**	10.48	1.01	10.50	0.94	10.52	1.08	10.60	1.08	0.06	0.4617	-0.58	1.32	-0.89	1.42	-0.87	1.76	-1.11	1.85	-0.10
RELATIVE LIPOPROTEIN LIPID CONCENTRATIONS	Free cholesterol to total lipids ratio in very large VLDL 1,5	XL-VLDL-FC %	%	10.15	2.53	9.76	2.61	9.99	1.65	9.83	1.82	-0.02	0.8285	11.59	1.38	11.71	1.56	11.10	1.68	11.55	2.35	-0.18	**0.0258**	1.57	2.30	1.34	2.42	0.91	2.32	1.33	2.27	-0.08
RELATIVE LIPOPROTEIN LIPID CONCENTRATIONS	Free cholesterol to total lipids ratio in very small VLDL	XS-VLDL-FC %	%	15.27	0.50	15.32	0.41	15.31	0.53	15.30	0.37	0.01	0.9379	15.72	0.35	15.71	0.29	15.72	0.41	15.68	0.34	-0.04	0.5954	0.44	0.48	0.43	0.37	0.35	0.51	0.37	0.42	-0.08
RELATIVE LIPOPROTEIN LIPID CONCENTRATIONS	Phospholipids to total lipids ratio in chylomicrons and extremely large VLDL* 5	XXL-VLDL-PL %	%											16.87	5.19	17.69	4.62	15.69	4.10	16.33	4.28	-0.17	**0.0451**									
RELATIVE LIPOPROTEIN LIPID CONCENTRATIONS	Phospholipids to total lipids ratio in IDL	IDL-PL %	%	22.55	1.37	22.58	0.95	22.71	1.32	22.74	0.95	0.07	0.3965	22.69	1.09	22.61	0.81	22.62	1.23	22.51	0.91	-0.05	0.5770	0.03	1.30	0.02	0.89	-0.18	1.09	-0.24	1.17	-0.15
RELATIVE LIPOPROTEIN LIPID CONCENTRATIONS	Phospholipids to total lipids ratio in large HDL	L-HDL-PL %	%	49.22	1.73	48.80	1.58	49.79	2.79	49.14	2.05	0.14	0.1039	47.42	1.57	47.54	1.39	47.57	1.91	47.94	1.67	0.11	0.1674	-1.38	1.59	-1.67	1.47	-1.50	2.26	-1.83	1.91	-0.03
RELATIVE LIPOPROTEIN LIPID CONCENTRATIONS	Phospholipids to total lipids ratio in large LDL	L-LDL-PL %	%	21.38	0.93	21.42	0.79	21.33	0.87	21.19	0.72	-0.10	0.2764	20.68	0.68	20.63	0.54	20.68	0.58	20.58	0.55	-0.05	0.5425	-0.84	0.90	-0.75	0.69	-0.66	1.08	-0.66	0.73	0.09
RELATIVE LIPOPROTEIN LIPID CONCENTRATIONS	Phospholipids to total lipids ratio in large VLDL	L-VLDL-PL %	%	17.47	3.10	18.41	4.70	18.23	2.13	18.95	3.17	0.09	0.3035	22.56	2.81	22.92	3.13	22.73	3.03	23.25	3.83	-0.01	0.9000	4.10	3.49	5.48	5.11	4.20	4.69	5.22	5.14	-0.07
RELATIVE LIPOPROTEIN LIPID CONCENTRATIONS	Phospholipids to total lipids ratio in medium HDL	M-HDL-PL %	%	45.78	1.04	45.69	0.78	46.09	1.07	46.14	0.87	0.21	**0.0161**	45.96	1.22	46.05	0.97	46.25	1.49	46.47	1.15	0.21	**0.0103**	0.22	1.12	0.29	0.87	0.43	1.54	0.38	1.08	0.06
RELATIVE LIPOPROTEIN LIPID CONCENTRATIONS	Phospholipids to total lipids ratio in medium LDL	M-LDL-PL %	%	26.91	1.12	27.10	0.90	26.63	1.05	26.70	0.87	-0.24	**0.0057**	25.79	0.77	25.70	0.64	25.74	0.77	25.58	0.66	-0.13	0.1101	-1.24	1.11	-1.20	1.02	-1.05	0.72	-1.08	0.87	0.08

				1ST TRIMESTER (N=185)										DELIVERY (N=201)										CHANGE BETWEEN DELIVERY AND 1ST TRIMESTER (DELTA) (N=150)								
				CONTROL (N=115)				SSRI (N=70)						CONTROL (N=112)				SSRI (N=89)						CONTROL (N=110)				SSRI (N=40)				
GROUP	VARIABLE	ABBRE-VIATION	UNIT	MEDIAN	IQR	MEAN	SD	MEDIAN	IQR	MEAN	SD	r	p	MEDIAN	IQR	MEAN	SD	MEDIAN	IQR	MEAN	SD	r	p	MEDIAN	IQR	MEAN	SD	MEDIAN	IQR	MEAN	SD	r
RELATIVE LIPOPROTEIN LIPID CONCENTRATIONS	Phospholipids to total lipids ratio in medium VLDL	M-VLDL-PL %	%	21.26	2.71	21.26	2.29	21.12	2.54	20.79	1.63	-0.08	0.3887	23.09	2.17	23.03	1.69	22.69	2.71	22.88	2.45	-0.09	0.2538	1.75	3.19	1.72	2.31	1.10	2.47	1.65	2.36	-0.05
RELATIVE LIPOPROTEIN LIPID CONCENTRATIONS	Phospholipids to total lipids ratio in small HDL	S-HDL-PL %	%	57.92	1.47	57.91	1.18	57.71	1.34	57.86	1.06	-0.07	0.4013	57.44	2.06	57.46	1.76	57.52	1.36	57.60	1.49	0.03	0.7575	-0.24	1.46	-0.43	1.55	-0.32	1.44	-0.24	1.28	0.01
RELATIVE LIPOPROTEIN LIPID CONCENTRATIONS	Phospholipids to total lipids ratio in small LDL	S-LDL-PL %	%	32.35	1.53	32.47	1.31	31.79	1.84	31.73	1.56	-0.27	**0.0018**	31.12	1.11	31.02	0.93	31.00	1.38	30.99	1.26	-0.03	0.7042	-1.35	1.91	-1.33	1.50	-1.09	1.84	-0.81	1.50	0.23
RELATIVE LIPOPROTEIN LIPID CONCENTRATIONS	Phospholipids to total lipids ratio in small VLDL	S-VLDL-PL %	%	22.17	2.61	22.19	1.83	22.16	2.01	21.81	1.63	-0.04	0.6333	21.96	1.85	21.89	1.36	21.52	1.56	21.49	1.45	-0.20	**0.0127**	-0.41	2.08	-0.33	1.61	-0.42	1.83	-0.61	1.47	-0.07
RELATIVE LIPOPROTEIN LIPID CONCENTRATIONS	Phospholipids to total lipids ratio in very large HDL	XL-HDL-PL %	%	49.82	2.38	50.78	3.31	48.69	3.67	49.83	3.60	-0.26	**0.0032**	50.03	2.30	49.57	2.31	49.35	2.70	49.11	2.59	-0.12	0.1407	-0.36	1.84	-0.28	2.44	-0.21	2.10	0.13	3.78	0.05
RELATIVE LIPOPROTEIN LIPID CONCENTRATIONS	Phospholipids to total lipids ratio in very large VLDL [1,5]	XL-VLDL-PL %	%	14.67	3.95	15.58	3.68	15.11	3.33	15.90	3.54	0.11	0.2300	19.61	1.42	19.73	1.61	19.23	1.68	19.59	2.11	-0.12	0.1495	4.21	3.46	4.87	3.42	3.01	3.17	3.94	3.94	-0.22
RELATIVE LIPOPROTEIN LIPID CONCENTRATIONS	Phospholipids to total lipids ratio in very small VLDL	XS-VLDL-PL %	%	28.61	1.23	28.73	1.03	28.69	1.19	28.83	1.02	0.05	0.5761	30.54	0.94	30.51	0.76	30.66	1.09	30.62	0.81	0.11	0.1782	1.76	1.12	1.92	0.98	1.99	1.21	1.95	1.06	0.05
RELATIVE LIPOPROTEIN LIPID CONCENTRATIONS	Cholesterol to total lipids ratio in chylomicrons and extremely large VLDL* [5]	XXL-VLDL-C %	%											28.99	17.77	36.57	18.27	26.17	12.17	32.33	17.52	-0.19	**0.0200**									
RELATIVE LIPOPROTEIN LIPID CONCENTRATIONS	Cholesterol to total lipids ratio in IDL	IDL-C %	%	68.04	2.50	68.33	1.89	67.65	2.69	67.96	2.03	-0.10	0.2487	65.01	3.23	64.88	2.36	64.10	3.28	63.90	2.87	-0.22	**0.0081**	-3.13	2.78	-3.16	2.15	-3.73	3.12	-3.97	2.74	-0.17
RELATIVE LIPOPROTEIN LIPID CONCENTRATIONS	Cholesterol to total lipids ratio in large HDL	L-HDL-C %	%	46.46	2.48	46.99	2.29	45.29	3.97	46.64	3.35	-0.19	**0.0331**	44.99	3.68	44.59	2.66	44.32	3.83	43.45	3.89	-0.18	**0.0314**	-1.62	2.93	-1.92	2.44	-1.69	3.01	-1.89	3.67	0.01
RELATIVE LIPOPROTEIN LIPID CONCENTRATIONS	Cholesterol to total lipids ratio in large LDL	L-LDL-C %	%	71.17	1.38	71.29	1.04	71.28	1.27	71.35	1.07	0.06	0.5195	69.51	2.17	69.24	1.69	68.79	2.25	68.50	1.92	-0.23	**0.0052**	-1.78	1.87	-1.95	1.62	-2.93	2.47	-2.97	2.08	-0.34
RELATIVE LIPOPROTEIN LIPID CONCENTRATIONS	Cholesterol to total lipids ratio in large VLDL	L-VLDL-C %	%	26.27	6.18	26.06	6.14	25.67	4.08	25.28	3.73	-0.09	0.3183	30.29	6.59	30.96	5.37	29.09	6.30	30.80	7.51	-0.10	0.2227	3.50	8.89	4.54	7.38	3.31	9.07	5.27	8.78	0.00
RELATIVE LIPOPROTEIN LIPID CONCENTRATIONS	Cholesterol to total lipids ratio in medium HDL	M-HDL-C %	%	49.52	2.27	49.61	1.83	48.80	2.25	48.87	2.05	-0.22	**0.0111**	45.29	4.06	44.82	3.50	44.24	4.98	43.85	3.54	-0.18	**0.0267**	-4.29	3.55	-4.77	3.20	-4.05	4.41	-4.89	2.98	-0.04
RELATIVE LIPOPROTEIN LIPID CONCENTRATIONS	Cholesterol to total lipids ratio in medium LDL	M-LDL-C %	%	66.03	2.09	66.21	1.79	66.55	2.32	66.67	1.73	0.17	**0.0493**	65.40	2.24	65.18	1.59	64.84	2.22	64.70	1.75	-0.16	**0.0452**	-0.84	2.08	-0.88	1.64	-2.10	2.24	-1.75	1.98	-0.32
RELATIVE LIPOPROTEIN LIPID CONCENTRATIONS	Cholesterol to total lipids ratio in medium VLDL	M-VLDL-C %	%	26.30	9.21	26.90	6.62	25.32	7.93	24.53	5.78	-0.10	0.2395	26.91	7.45	26.98	5.45	25.21	7.73	25.72	6.83	-0.15	0.0637	0.54	8.64	0.47	6.59	-1.02	8.13	0.26	6.61	-0.03
RELATIVE LIPOPROTEIN LIPID CONCENTRATIONS	Cholesterol to total lipids ratio in small HDL	S-HDL-C %	%	37.94	2.06	38.25	1.86	37.84	1.97	37.87	1.58	-0.11	0.2113	34.66	3.10	34.50	2.12	34.14	3.30	33.83	2.31	-0.17	**0.0419**	-3.44	2.80	-3.52	1.91	-3.63	2.86	-3.88	2.25	-0.06
RELATIVE LIPOPROTEIN LIPID CONCENTRATIONS	Cholesterol to total lipids ratio in small LDL	S-LDL-C %	%	61.49	2.25	61.67	1.71	62.01	2.35	62.30	1.68	0.20	**0.0196**	60.68	2.69	60.37	1.92	59.75	2.69	59.68	2.22	-0.20	**0.0161**	-1.01	2.51	-1.16	1.79	-2.47	2.82	-2.28	2.74	-0.29
RELATIVE LIPOPROTEIN LIPID CONCENTRATIONS	Cholesterol to total lipids ratio in small VLDL	S-VLDL-C %	%	31.99	6.17	32.02	4.63	32.35	4.00	31.73	3.96	-0.01	0.9425	34.84	4.65	34.52	3.52	33.67	5.38	33.55	4.03	-0.16	0.0501	2.24	5.38	2.42	4.33	1.71	4.84	1.38	4.34	-0.11
RELATIVE LIPOPROTEIN LIPID CONCENTRATIONS	Cholesterol to total lipids ratio in very large HDL	XL-HDL-C %	%	46.39	2.23	45.81	2.96	46.75	2.99	45.99	2.75	0.08	0.3779	43.35	1.73	43.47	1.59	42.92	1.78	42.98	1.96	-0.15	0.0745	-2.38	2.29	-2.93	2.51	-2.66	3.18	-3.62	3.44	-0.08
RELATIVE LIPOPROTEIN LIPID CONCENTRATIONS	Cholesterol to total lipids ratio in very large VLDL[1,5]	XL-VLDL-C %	%	28.04	10.99	25.80	11.48	26.19	8.62	23.37	9.90	-0.11	0.2262	25.74	6.92	26.65	6.11	23.76	6.37	26.04	9.13	-0.19	**0.0210**	0.14	11.74	-2.38	11.07	-0.25	8.91	-0.37	8.11	0.06
RELATIVE LIPOPROTEIN LIPID CONCENTRATIONS	Cholesterol to total lipids ratio in very small VLDL	XS-VLDL-C %	%	48.70	4.17	48.65	2.93	48.21	4.66	48.15	3.36	-0.10	0.2702	44.93	3.86	44.68	3.04	43.79	3.74	43.51	3.69	-0.22	**0.0088**	-4.31	3.96	-4.09	3.15	-4.83	5.14	-4.83	3.55	-0.12
RELATIVE LIPOPROTEIN LIPID CONCENTRATIONS	Cholesteryl esters to total lipids ratio in chylomicrons and extremely large VLDL*[5]	XXL-VLDL-CE %	%											17.73	12.31	23.12	13.95	16.13	8.69	20.13	13.35	-0.18	**0.0295**									
RELATIVE LIPOPROTEIN LIPID CONCENTRATIONS	Cholesteryl esters to total lipids ratio in IDL	IDL-CE %	%	51.02	2.32	51.12	1.71	50.82	2.55	50.94	1.83	-0.04	0.6193	48.61	2.75	48.64	1.97	47.78	2.75	47.96	2.27	-0.20	**0.0164**	-2.33	2.75	-2.36	1.92	-2.89	2.08	-2.98	2.32	-0.15
RELATIVE LIPOPROTEIN LIPID CONCENTRATIONS	Cholesteryl esters to total lipids ratio in large HDL	L-HDL-CE %	%	35.91	2.16	36.55	2.04	34.88	3.75	36.02	2.88	-0.20	**0.0233**	33.88	3.38	33.47	2.66	33.04	4.05	32.42	3.65	-0.18	**0.0274**	-1.97	2.58	-2.48	2.46	-2.13	3.03	-2.41	3.37	0.03
RELATIVE LIPOPROTEIN LIPID CONCENTRATIONS	Cholesteryl esters to total lipids ratio in large LDL	L-LDL-CE %	%	52.36	1.75	52.29	1.24	52.75	1.16	52.97	1.29	0.22	**0.0117**	52.06	1.43	52.04	1.15	51.93	1.77	51.78	1.43	-0.07	0.4170	-0.27	2.05	-0.33	1.43	-1.19	1.67	-1.05	1.46	-0.29
RELATIVE LIPOPROTEIN LIPID CONCENTRATIONS	Cholesteryl esters to total lipids ratio in large VLDL	L-VLDL-CE %	%	14.03	4.37	13.84	4.24	13.24	2.55	12.90	2.70	-0.14	0.1158	15.87	4.71	16.28	3.68	15.05	4.24	16.09	5.14	-0.11	0.1970	1.58	6.50	2.14	5.01	1.72	5.30	2.94	6.08	0.04
RELATIVE LIPOPROTEIN LIPID CONCENTRATIONS	Cholesteryl esters to total lipids ratio in medium HDL	M-HDL-CE %	%	40.28	2.13	40.52	1.78	39.66	2.13	39.76	1.88	-0.22	**0.0107**	35.34	3.78	34.87	3.56	34.82	5.30	34.02	3.51	-0.16	0.0549	-5.13	3.58	-5.46	3.28	-4.94	4.29	-5.49	3.05	-0.03
RELATIVE LIPOPROTEIN LIPID CONCENTRATIONS	Cholesteryl esters to total lipids ratio in medium LDL	M-LDL-CE %	%	45.88	2.77	45.95	2.18	46.87	2.83	47.04	2.41	0.27	**0.0018**	48.04	1.96	48.14	1.54	48.21	2.45	48.32	2.01	0.08	0.3568	2.32	2.54	2.28	2.15	1.44	2.08	1.64	2.12	-0.19
RELATIVE LIPOPROTEIN LIPID CONCENTRATIONS	Cholesteryl esters to total lipids ratio in medium VLDL	M-VLDL-CE %	%	13.79	6.91	14.24	4.88	12.93	6.55	12.67	4.43	-0.12	0.1720	13.30	5.65	13.34	4.06	11.90	5.66	12.35	4.94	-0.15	0.0606	-0.50	6.15	-0.62	4.86	-0.99	6.13	-0.64	4.76	-0.01
RELATIVE LIPOPROTEIN LIPID CONCENTRATIONS	Cholesteryl esters to total lipids ratio in small HDL	S-HDL-CE %	%	27.13	2.53	27.64	2.19	27.10	2.29	27.16	1.84	-0.06	0.4678	21.94	4.01	21.87	2.70	21.41	4.09	21.26	2.99	-0.11	0.1727	-5.05	3.46	-5.35	2.39	-5.53	3.27	-5.65	2.88	-0.06
RELATIVE LIPOPROTEIN LIPID CONCENTRATIONS	Cholesteryl esters to total lipids ratio in small LDL	S-LDL-CE %	%	43.60	1.66	43.58	1.45	44.30	2.15	44.28	1.89	0.27	**0.0024**	45.10	1.45	45.23	1.17	45.08	1.97	45.01	1.82	-0.05	0.5425	1.64	2.03	1.63	1.77	1.37	3.33	0.70	2.35	-0.24
RELATIVE LIPOPROTEIN LIPID CONCENTRATIONS	Cholesteryl esters to total lipids ratio in small VLDL	S-VLDL-CE %	%	19.26	4.13	19.57	2.98	19.66	3.54	19.23	2.43	0.02	0.7956	22.25	3.10	22.01	2.34	21.72	3.42	21.52	2.73	-0.10	0.2136	2.52	3.17	2.69	2.86	2.27	3.74	2.03	2.97	-0.09

				1ST TRIMESTER (N=185)										DELIVERY (N=201)										CHANGE BETWEEN DELIVERY AND 1ST TRIMESTER (DELTA) (N=150)								
				CONTROL (N=115)				SSRI (N=70)						CONTROL (N=112)				SSRI (N=89)						CONTROL (N=110)				SSRI (N=40)				
GROUP	VARIABLE	ABBRE-VIATION	UNIT	MEDIAN	IQR	MEAN	SD	MEDIAN	IQR	MEAN	SD	r	p	MEDIAN	IQR	MEAN	SD	MEDIAN	IQR	MEAN	SD	r	p	MEDIAN	IQR	MEAN	SD	MEDIAN	IQR	MEAN	SD	r
RELATIVE LIPOPROTEIN LIPID CONCENTRATIONS	Cholesteryl esters to total lipids ratio in very large HDL	XL-HDL-CE %	%	34.98	1.89	34.90	1.83	34.85	1.95	34.80	1.66	-0.04	0.6598	32.85	1.80	32.97	1.44	32.43	2.20	32.38	1.81	-0.19	**0.0202**	-1.82	1.85	-2.04	1.68	-1.93	2.54	-2.51	2.38	-0.08
RELATIVE LIPOPROTEIN LIPID CONCENTRATIONS	Cholesteryl esters to total lipids ratio in very large VLDL [5]	XL-VLDL-CE %	%	17.89	9.28	15.56	9.37	16.20	7.68	14.20	8.76	-0.13	0.1423	14.13	6.09	14.94	4.62	12.72	4.65	14.48	6.86	-0.18	**0.0253**	-1.24	9.92	-3.72	9.14	-0.61	7.45	-1.70	6.92	0.11
RELATIVE LIPOPROTEIN LIPID CONCENTRATIONS	Cholesteryl esters to total lipids ratio in very small VLDL	XS-VLDL-CE %	%	33.42	3.81	33.46	2.69	32.90	4.18	32.90	3.15	-0.10	0.2361	29.23	3.59	28.97	2.93	28.15	3.93	27.83	3.55	-0.22	**0.0083**	-4.84	3.78	-4.52	2.96	-5.03	4.72	-5.21	3.34	-0.12
RELATIVE LIPOPROTEIN LIPID CONCENTRATIONS (LARGE HDL RATIOS)	Triglycerides to total lipids ratio in chylomicrons and extremely large VLDL[*5]	XXL-VLDL-TG %	%											53.96	22.93	45.74	20.90	57.68	16.20	51.34	19.05	0.18	**0.0260**									
RELATIVE LIPOPROTEIN LIPID CONCENTRATIONS (LARGE HDL RATIOS)	Triglycerides to total lipids ratio in IDL	IDL-TG %	%	9.41	1.98	9.16	1.51	9.63	2.47	9.59	1.61	0.09	0.2995	11.95	2.84	12.52	2.25	13.30	3.22	13.59	2.99	0.24	**0.0030**	2.90	2.19	3.14	2.00	3.88	3.44	4.21	3.12	0.22
RELATIVE LIPOPROTEIN LIPID CONCENTRATIONS (LARGE HDL RATIOS)	Triglycerides to total lipids ratio in large HDL	L-HDL-TG %	%	4.32	1.40	4.09	1.20	4.92	2.01	4.59	1.66	0.21	**0.0150**	7.39	2.71	7.87	2.46	8.10	2.91	8.62	2.95	0.18	**0.0301**	3.17	2.32	3.59	2.32	2.72	2.73	3.72	2.57	0.00
RELATIVE LIPOPROTEIN LIPID CONCENTRATIONS (LARGE HDL RATIOS)	Triglycerides to total lipids ratio in large LDL	L-LDL-TG %	%	7.46	1.33	7.14	1.29	7.39	1.42	7.43	1.15	0.01	0.9110	9.96	2.33	10.13	1.89	10.77	2.48	10.92	2.19	0.23	**0.0045**	2.61	1.79	2.71	1.63	3.65	2.59	3.63	2.24	0.30
RELATIVE LIPOPROTEIN LIPID CONCENTRATIONS (LARGE HDL RATIOS)	Triglycerides to total lipids ratio in large VLDL	L-VLDL-TG %	%	56.25	8.00	56.37	9.84	56.10	5.32	55.86	5.99	0.01	0.9334	46.72	8.89	46.12	7.63	48.61	9.57	45.95	10.83	0.06	0.4368	-7.81	12.29	-10.02	11.15	-6.46	13.62	-10.49	13.41	0.04
RELATIVE LIPOPROTEIN LIPID CONCENTRATIONS (LARGE HDL RATIOS)	Triglycerides to total lipids ratio in medium HDL	M-HDL-TG %	%	4.69	1.47	4.54	1.28	5.11	1.45	5.05	1.35	0.21	**0.0193**	8.71	3.00	9.13	2.77	9.22	3.63	9.68	2.69	0.15	0.0730	4.06	2.89	4.48	2.56	4.28	2.95	4.50	2.29	0.05
RELATIVE LIPOPROTEIN LIPID CONCENTRATIONS (LARGE HDL RATIOS)	Triglycerides to total lipids ratio in medium LDL	M-LDL-TG %	%	7.07	1.60	6.70	1.73	6.82	1.87	6.75	1.34	-0.05	0.5838	8.86	2.29	9.12	1.70	9.65	2.20	9.73	1.92	0.21	**0.0122**	1.93	1.83	2.08	1.49	2.87	2.45	2.84	1.85	0.28
RELATIVE LIPOPROTEIN LIPID CONCENTRATIONS (LARGE HDL RATIOS)	Triglycerides to total lipids ratio in medium VLDL	M-VLDL-TG %	%	52.44	12.06	51.61	8.75	53.56	11.44	54.54	7.27	0.10	0.2641	50.03	9.30	49.99	7.10	51.97	10.37	51.39	9.20	0.14	0.0942	-1.97	11.88	-2.19	8.76	-0.03	10.15	-1.91	8.84	0.03
RELATIVE LIPOPROTEIN LIPID CONCENTRATIONS (LARGE HDL RATIOS)	Triglycerides to total lipids ratio in small HDL	S-HDL-TG %	%	4.14	1.23	4.00	1.07	4.45	1.25	4.48	1.01	0.20	**0.0216**	7.77	2.31	8.04	2.11	8.46	3.11	8.57	2.20	0.16	0.0521	3.75	2.26	3.94	1.87	4.19	2.26	4.11	1.89	0.09
RELATIVE LIPOPROTEIN LIPID CONCENTRATIONS (LARGE HDL RATIOS)	Triglycerides to total lipids ratio in small LDL	S-LDL-TG %	%	6.16	1.53	5.72	1.76	6.21	1.70	6.11	1.29	0.12	0.1641	8.41	2.19	8.61	1.71	9.11	2.62	9.33	1.97	0.25	**0.0028**	2.54	2.00	2.49	1.60	3.09	2.23	3.09	1.80	0.18
RELATIVE LIPOPROTEIN LIPID CONCENTRATIONS (LARGE HDL RATIOS)	Triglycerides to total lipids ratio in small VLDL	S-VLDL-TG %	%	45.84	8.68	45.97	6.36	45.49	5.21	46.52	5.51	0.02	0.8219	43.41	6.04	43.59	4.79	45.01	6.67	44.97	5.35	0.18	**0.0279**	-1.87	7.38	-2.09	5.84	-1.01	6.49	-0.77	5.65	0.10
RELATIVE LIPOPROTEIN LIPID CONCENTRATIONS (LARGE HDL RATIOS)	Triglycerides to total lipids ratio in very large HDL	XL-HDL-TG %	%	3.79	1.43	3.54	1.24	4.56	2.22	4.01	1.92	0.22	**0.0119**	6.74	2.69	6.97	2.17	7.49	3.00	7.92	3.45	0.19	**0.0199**	3.03	2.18	3.21	2.01	2.71	2.44	3.49	3.31	-0.02
RELATIVE LIPOPROTEIN LIPID CONCENTRATIONS (LARGE HDL RATIOS)	Triglycerides to total lipids ratio in very large VLDL[1,5]	XL-VLDL-TG %	%	57.29	10.37	58.89	12.80	58.70	8.96	60.34	9.46	0.07	0.4567	54.48	7.63	53.63	7.51	56.71	7.48	54.37	10.94	0.18	**0.0253**	-3.44	13.14	-2.49	12.04	-3.09	10.29	-3.57	9.32	0.02
RELATIVE LIPOPROTEIN LIPID CONCENTRATIONS (LARGE HDL RATIOS)	Triglycerides to total lipids ratio in very small VLDL	XS-VLDL-TG %	%	22.70	3.50	22.39	2.75	23.10	4.75	23.43	3.06	0.10	0.2533	24.37	3.24	24.81	2.54	25.59	3.31	25.87	3.25	0.22	**0.0072**	2.40	3.51	2.17	2.83	2.87	4.97	2.88	3.33	0.12
TOTAL LIPIDS	Total lipids in HDL	HDL-L	mmol/l	4.02	0.86	4.11	0.56	3.87	0.80	3.96	0.64	-0.17	0.0548	4.58	1.05	4.58	0.84	4.47	1.05	4.48	0.80	-0.06	0.4383	0.55	0.92	0.56	0.76	0.49	0.98	0.47	0.64	-0.07
TOTAL LIPIDS	Total lipids in LDL	LDL-L	mmol/l	2.30	0.75	2.24	0.49	2.45	0.60	2.35	0.55	0.15	0.0814	3.86	1.07	3.92	0.90	3.67	0.99	3.82	0.89	-0.07	0.4100	1.60	0.97	1.62	0.78	1.45	0.88	1.28	0.76	-0.22
TOTAL LIPIDS	Total lipids in lipoprotein particles	Total-L	mmol/l	9.04	1.71	8.97	1.39	9.36	2.31	8.97	1.65	0.10	0.2498	14.59	3.43	14.60	2.55	14.14	4.01	14.64	2.71	-0.01	0.8710	5.34	3.04	5.57	2.38	4.83	3.10	4.91	2.23	-0.16
TRIGLYCERIDES	Total triglycerides	Total-TG	mmol/l	1.13	0.44	1.02	0.46	1.31	0.63	1.21	0.52	0.24	**0.0063**	2.71	1.29	2.79	0.99	2.84	1.70	3.08	1.20	0.14	0.1001	1.58	1.30	1.68	0.92	1.46	1.50	1.73	1.03	0.00
TRIGLYCERIDES	Triglycerides in HDL	HDL-TG	mmol/l	0.18	0.06	0.16	0.06	0.18	0.07	0.18	0.05	0.14	0.1198	0.37	0.11	0.36	0.08	0.38	0.12	0.38	0.09	0.10	0.2163	0.19	0.12	0.19	0.08	0.18	0.10	0.19	0.08	-0.02
TRIGLYCERIDES	Triglycerides in large LDL	L-LDL-TG	mmol/l	0.11	0.03	0.11	0.03	0.12	0.03	0.11	0.03	0.09	0.2852	0.25	0.08	0.25	0.06	0.25	0.09	0.26	0.07	0.07	0.3841	0.13	0.07	0.14	0.05	0.13	0.06	0.14	0.06	0.00
TRIGLYCERIDES	Triglycerides in VLDL	VLDL-TG	mmol/l	0.67	0.38	0.57	0.37	0.84	0.55	0.74	0.43	0.25	**0.0039**	1.65	1.00	1.79	0.82	1.87	1.39	2.03	1.02	0.14	0.0942	1.05	0.92	1.13	0.76	0.97	1.14	1.19	0.89	0.00

p < 0.05 bolded
[*] Values from 1st trimester and delta removed for having 59% missing values
[1] 1st trimester n = 167; [2] 1st trimester n = 184; [3] delivery n = 189; [4] delivery n = 195, [5] delivery n = 200
C , cholesterol; IDL , intermediate-density lipoprotein; IQR , interquartile range; LDL , low-density lipoprotein; SD , standard deviation; SSRI , selective serotonin reuptake inhibitor; VLDL , very low-density lipoprotein

Table S3. Linear regression model from 1st trimester timepoint with the NMR analysis variables as the dependent variable, body mass index (BMI) as a predictor and study group (selective serotonin reuptake inhibitor (SSRI)-case vs. control), gestational diabetes mellitus (GDM) and additional medication use as cofactors.

Group	Variable	Overall regression			SSRI medication		BMI		GDM		Additional medication use	
		Adjusted R^2	F	p	B	p	B	p	B	p	B	p
Lipoprotein subclasses	Cholesteryl esters in chylomicrons and extremely large VLDL (mmol/l)	0.132	7.61	**< 0.001**	0.139	**0.012**	0.016	**0.005**	0.062	0.349	0.056	0.283
Lipoprotein subclasses	Concentration of chylomicrons and extremely large VLDL particles (mmol/l)	0.086	5.09	**< 0.001**	0.138	**0.024**	0.014	**0.004**	0.049	0.506	0.055	0.343
Lipoprotein subclasses	Concentration of very large VLDL particles (mmol/l)	0.152	8.76	**< 0.001**	0.112	**0.016**	0.016	**< 0.001**	0.047	0.402	0.055	0.217
Lipoprotein subclasses	Free cholesterol in chylomicrons and extremely large VLDL (mmol/l)	0.107	6.19	**< 0.001**	0.130	**0.024**	0.016	**< 0.001**	0.051	0.451	0.048	0.378
Lipoprotein subclasses	Free cholesterol in large VLDL (mmol/l)	0.167	9.66	**< 0.001**	0.100	**0.017**	0.016	**< 0.001**	0.038	0.448	0.047	0.240
Lipoprotein subclasses	Free cholesterol in very large VLDL (mmol/l)	0.160	9.22	**< 0.001**	0.125	**0.010**	0.017	**< 0.001**	0.044	0.449	0.062	0.184
Lipoprotein subclasses	Phospholipids in chylomicrons and extremely large VLDL (mmol/l)	0.096	5.62	**< 0.001**	0.137	**0.025**	0.016	**0.001**	0.045	0.541	0.053	0.364
Lipoprotein subclasses	Phospholipids in very large VLDL (mmol/l)	0.151	8.67	**< 0.001**	0.131	**0.012**	0.017	**< 0.001**	0.047	0.457	0.066	0.187
Lipoprotein subclasses	Total lipids in chylomicrons and extremely large VLDL (mmol/l)	0.080	4.74	**0.001**	0.130	**0.031**	0.014	**0.005**	0.037	0.607	0.071	0.218
Lipoprotein subclasses	Total lipids in large VLDL (mmol/l)	0.133	7.62	**< 0.001**	0.097	**0.020**	0.013	**< 0.001**	0.039	0.437	0.048	0.227
Lipoprotein subclasses	Total lipids in very large VLDL (mmol/l)	0.152	8.76	**< 0.001**	0.125	**0.012**	0.016	**< 0.001**	0.045	0.443	0.069	0.145
Lipoprotein subclasses	Triglycerides in chylomicrons and extremely large VLDL (mmol/l)	0.058	3.68	**0.006**	0.124	0.048	0.012	**0.018**	0.029	0.696	0.080	0.180
Lipoprotein subclasses	Triglycerides in large VLDL (mmol/l)	0.114	6.56	**< 0.001**	0.100	**0.020**	0.012	**< 0.001**	0.043	0.401	0.049	0.231
Lipoprotein subclasses	Triglycerides in very large VLDL (mmol/l)	0.137	7.87	**< 0.001**	0.130	**0.013**	0.016	**< 0.001**	0.052	0.400	0.070	0.153
Lipoprotein subclasses	Cholesterol in chylomicrons and extremely large VLDL (mmol/l)	0.125	7.20	**< 0.001**	0.135	**0.015**	0.016	**< 0.001**	0.057	0.386	0.054	0.309
Lipoprotein subclasses	Cholesterol in very large VLDL (mmol/l)	0.182	10.61	**< 0.001**	0.108	**0.013**	0.017	**< 0.001**	0.03	0.565	0.062	0.139
Relative lipoprotein lipid	Phospholipids to total lipids ratio in small LDL (%)	0.261	16.31	**< 0.001**	-0.003	0.257	-0.001	**< 0.001**	-0.01	**0.006**	0.0002	0.953

Group	Variable	Overall regression			SSRI medication		BMI		GDM		Additional medication use	
		Adjusted R^2	F	p	B	p	B	p	B	p	B	p
concentrations												
Relative lipoprotein lipid concentrations	Cholesteryl esters to total lipids ratio in medium LDL (%)	0.193	11.40	**< 0.001**	0.006	0.119	0.002	**< 0.001**	0.001	0.850	0.002	0.486
Relative lipoprotein lipid concentrations	Cholesteryl esters to total lipids ratio in small LDL (%)	0.305	19.98	**< 0.001**	0.003	0.245	0.001	**< 0.001**	0.010	**0.002**	-0.001	0.551

BMI, Body Mass Index; GDM, Gestational diabetes mellitus; IDL, intermediate-density lipoprotein; LDL, low-density lipoprotein; SSRI, selective serotonin reuptake inhibitor; VLDL, very low-density lipoprotein. p< 0.05 bolded. Missing data in SSRI-cases, n=2 (BMI), Missing data in controls n=7 (BMI)

Table S4. Linear regression model from delivery timepoint with the NMR analysis variable as the dependent variable, body mass index (BMI) and cotinine level as predictors and study group (selective serotonin reuptake inhibitor (SSRI)-case vs. control), gestational diabetes mellitus (GDM) and additional medication use as cofactors.

Group	Variable	Overall regression			SSRI medication		BMI		GDM		Cotinine level (ng/ml)		Additional medication use	
		Adjusted R^2	F	p	B	p	B	p	B	p	B	p	B	p
Fatty acids (ratios)	Ratio of polyunsaturated fatty acids to total fatty acids (%)	0.060	3.29	**0.007**	-0.015	**0.028**	-0.001	0.242	-0.012	0.133	0	0.158	0	1.000
Relative lipoprotein lipid concentrations	Triglycerides to total lipids ratio in small LDL (%)	0.085	4.31	**0.001**	0.036	**0.014**	0.001	0.324	0.037	**0.034**	0.0006	0.117	0.006	0.674
Relative lipoprotein lipid concentrations	Triglycerides to total lipids ratio in IDL (%)	0.114	5.60	**< 0.001**	0.033	**0.021**	0.002	0.045	0.035	**0.039**	0.001	0.081	-0.003	0.802

IDL, intermediate-density lipoprotein; LDL, low-density lipoprotein; SSRI, selective serotonin reuptake inhibitor; VLDL, very low-density lipoprotein. p< 0.05 bolded. Missing data in controls n=6 (BMI, metabolite)

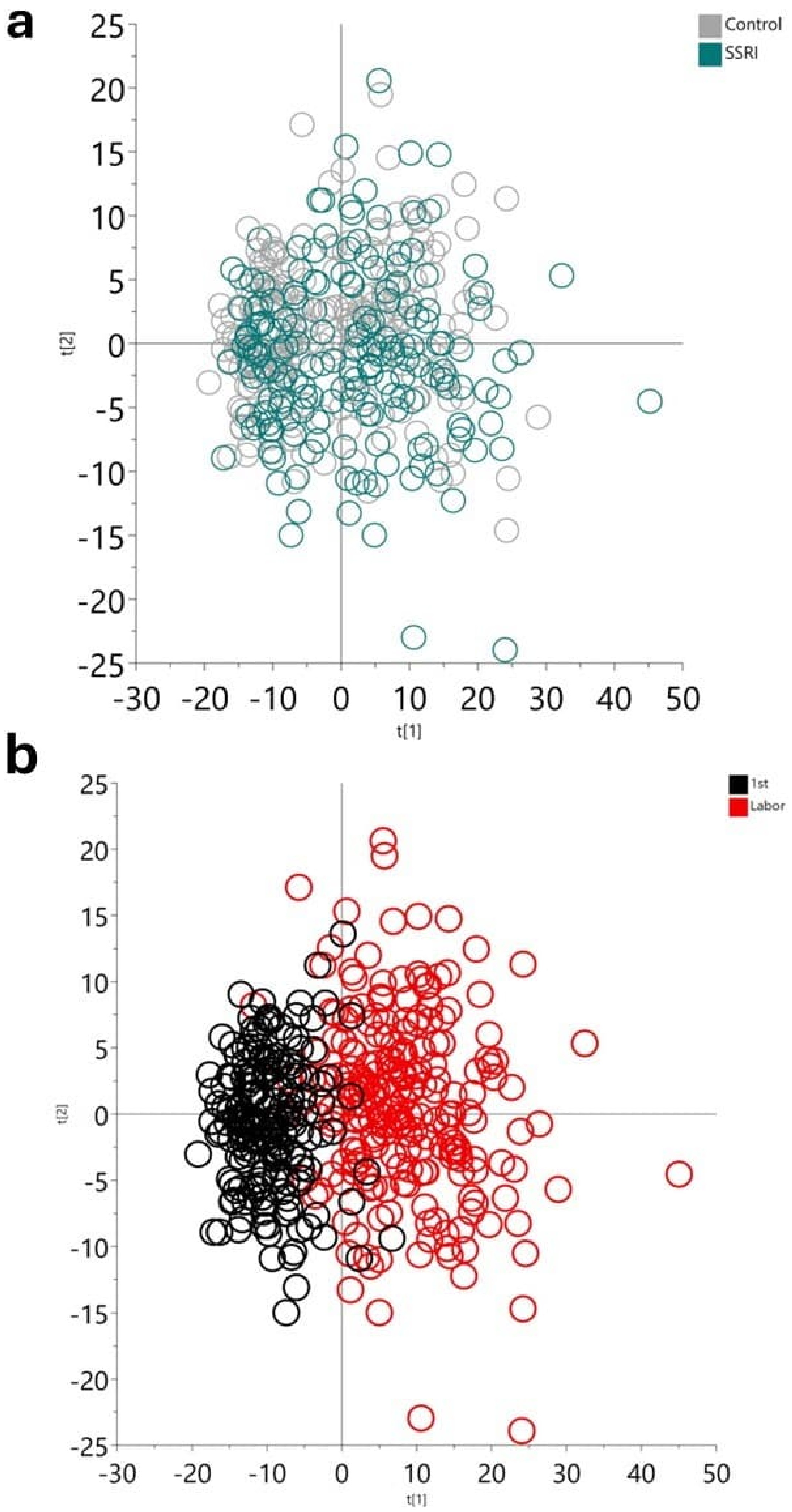

Fig S1 Principal component analysis (PCA) showing patterns of a metabolic profile between (a) SSRI users (n = 122) and controls (n = 117) and (b) 1st trimester and delivery timepoints of pregnancy (n= 239)

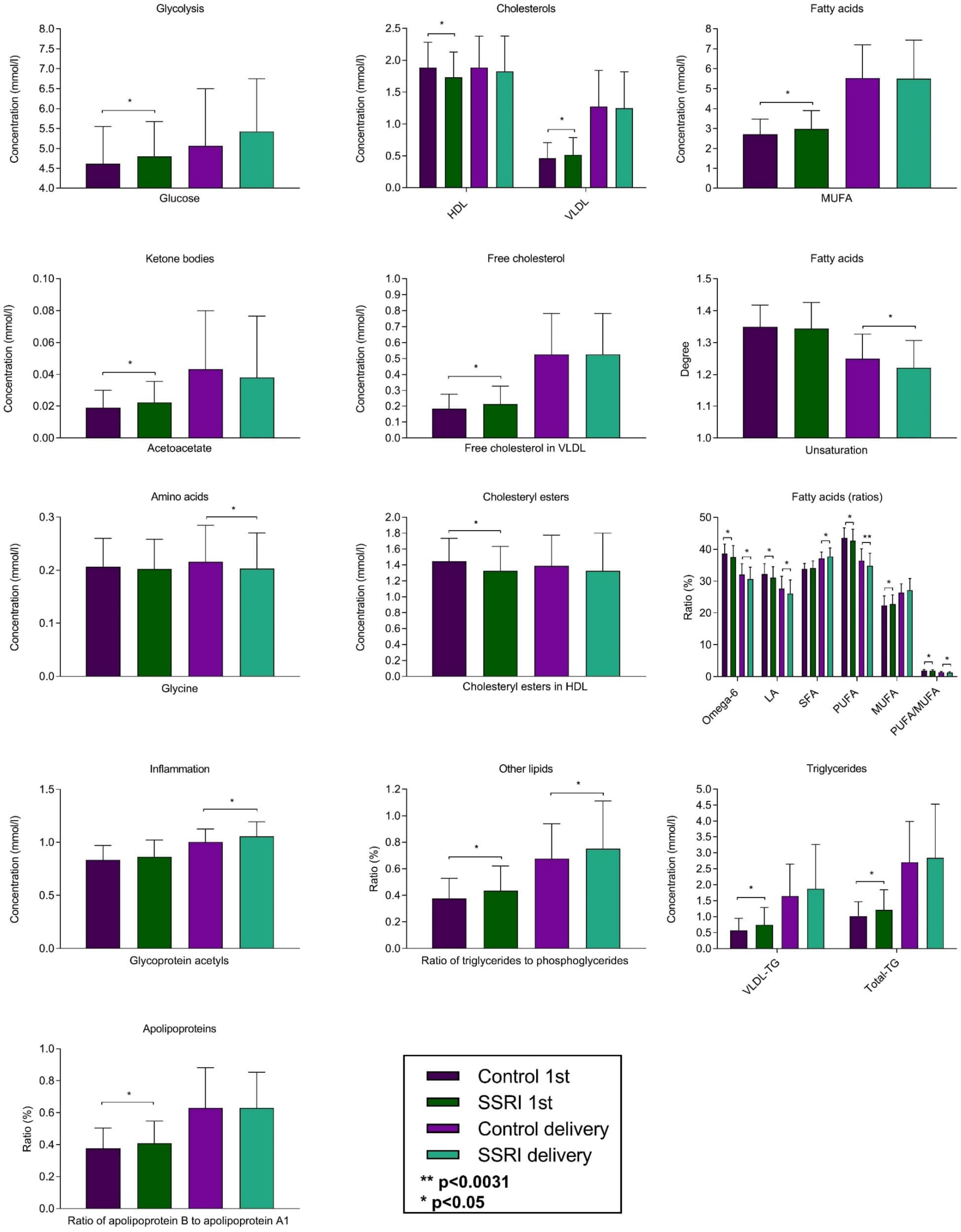

Fig S2 Comparison of the variable levels from the first trimester and delivery timepoints of pregnancy between SSRI-users (n=122) and control women (n=117). Wilcoxon rank-sum test was used to compare the variable levels between groups. Significant differences at the multiple testing corrected α-level ($p < 0.0031$) and the nominal p-value < 0.05 are represented. Error bars represent the median value and interquartile range (IQR). HDL, high-density lipoprotein; LDL, low-density lipoprotein; MUFA, Monounsaturated fatty acids; Omega-6, ratio of omega-6 fatty acids to total fatty acids; LA, ratio of linoleic acid to total fatty acids; SFA, saturated fatty acids; PUFA, ratio of polyunsaturated fatty acids to total fatty acids; MUFA %, ratio of monounsaturated fatty acids to total fatty acids; PUFA/MUFA, ratio of polyunsaturated fatty acids to monounsaturated fatty acids; VLDL-TG, Triglycerides in VLDL; Total TG, Total triglycerides

Lipoprotein subclasses

Relative lipoprotein lipid concentrations

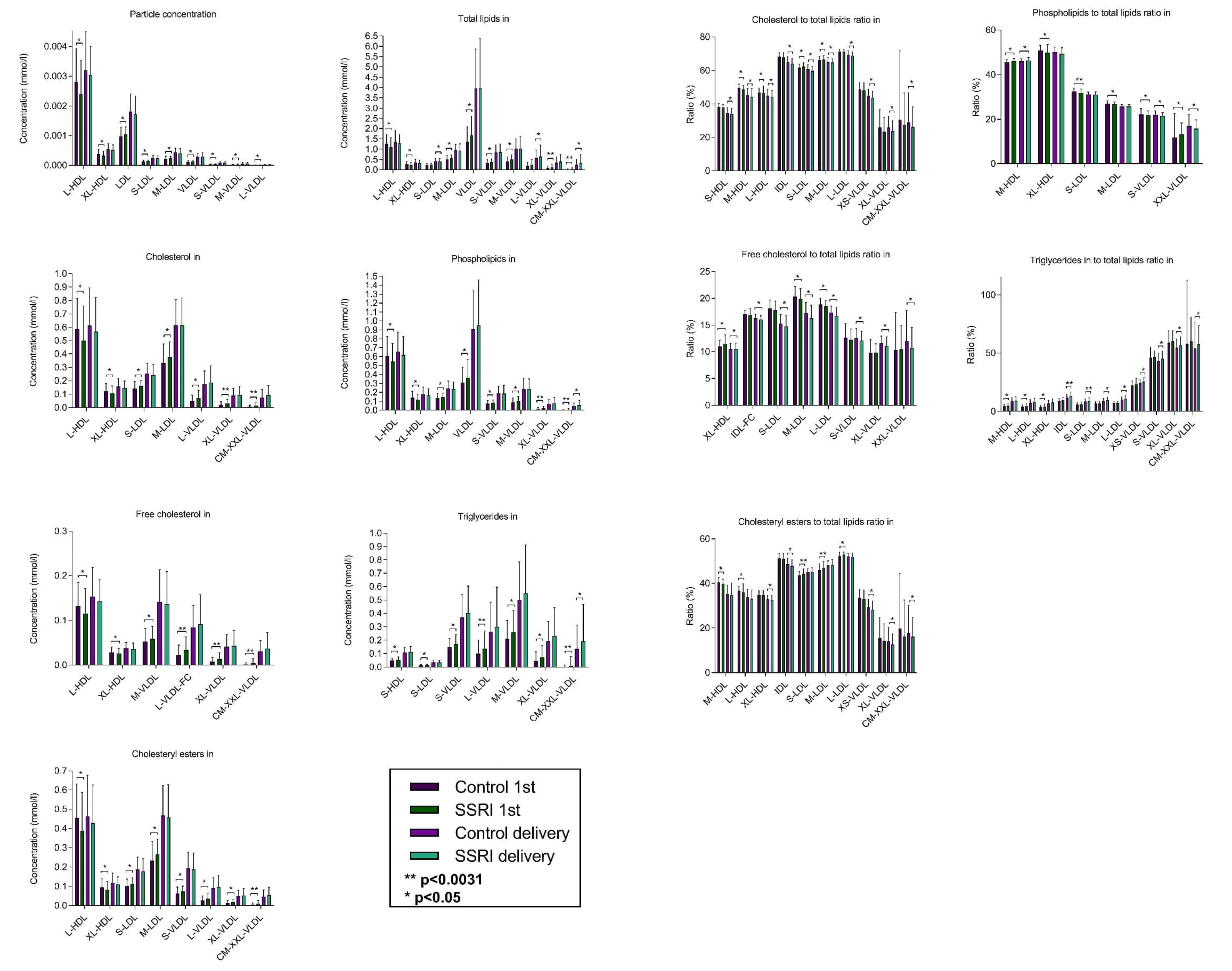

Fig S3 Comparison of the variable levels from the first trimester and delivery timepoints of pregnancy between SSRI-users (n=122) and control women (n=117). Wilcoxon rank-sum test was used to compare the variable levels between groups. Significant differences at the multiple testing corrected α-level ($p < 0.0031$) and the nominal p-value < 0.05 are represented. Error bars represent the median value and interquartile range (IQR). XS, very small; S, small; M, medium; L, large; XL, very large; XXL, extremely large; CM, chylomicron; VLDL, very low-density lipoprotein; LDL, low-density lipoprotein; IDL, intermediate-density lipoprotein; HDL, high-density lipoprotein

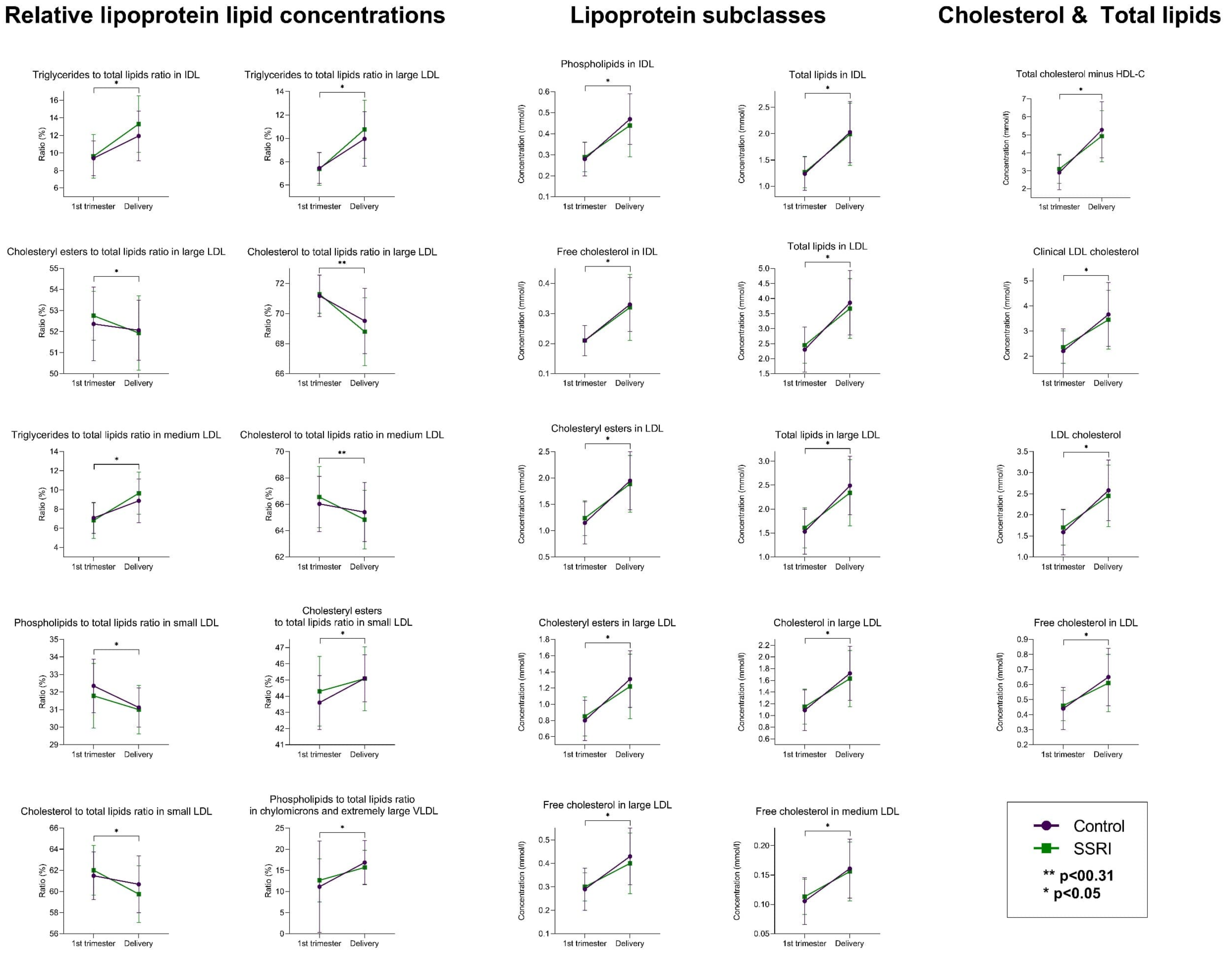

Fig S4 Comparison of the changes on the metabolic variables during pregnancy between SSRI-users (n=37) and control women (n=113).

Wilcoxon rank-sum test was used to compare the metabolite levels between groups (n = 150). Significant differences with the nominal p-value < 0.05 are represented.

Error bars represent the median value and interquartile range (IQR)

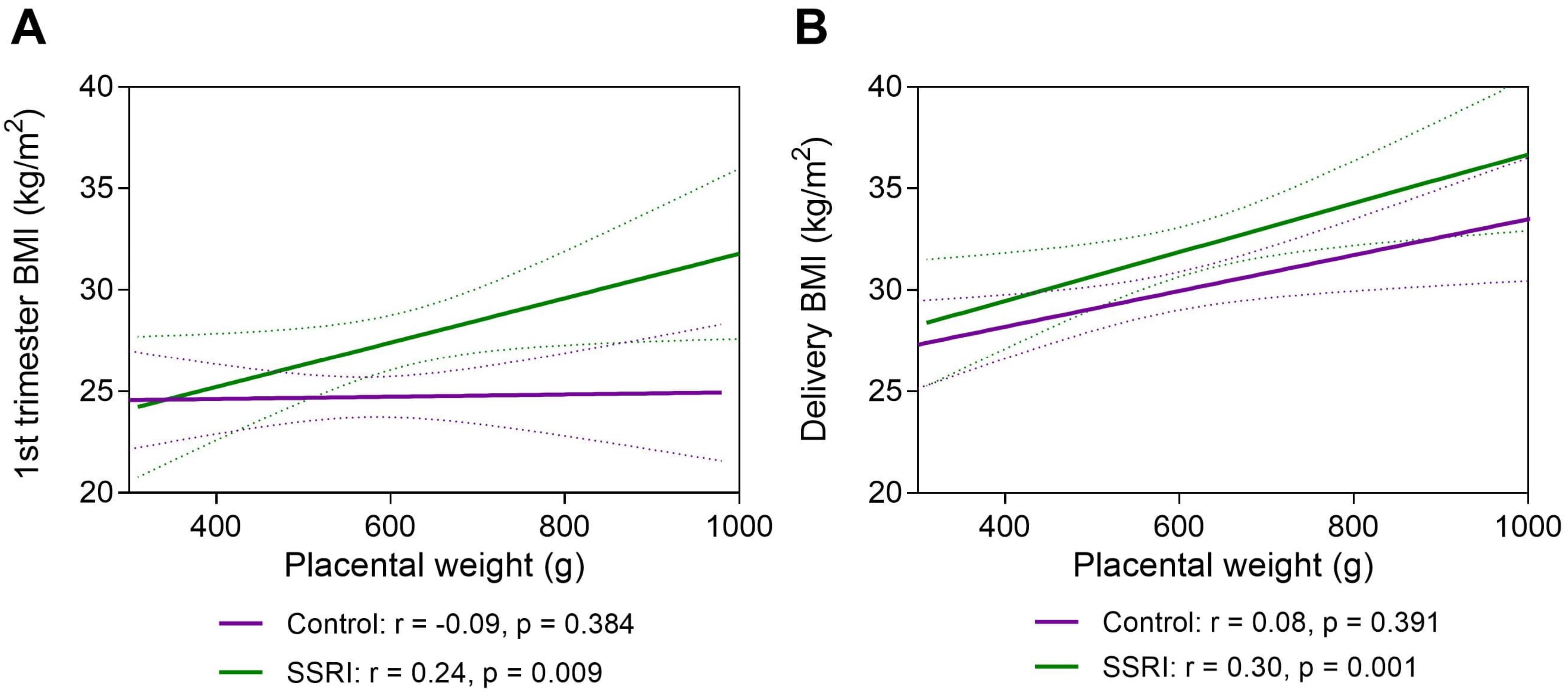

Fig S5 Spearman's correlation with 95 % confidence interval between placental weight and **(**a) 1st trimester body mass index (BMI) and **(**b) BMI at delivery in SSRI users (n = 122) and controls (n = 117)

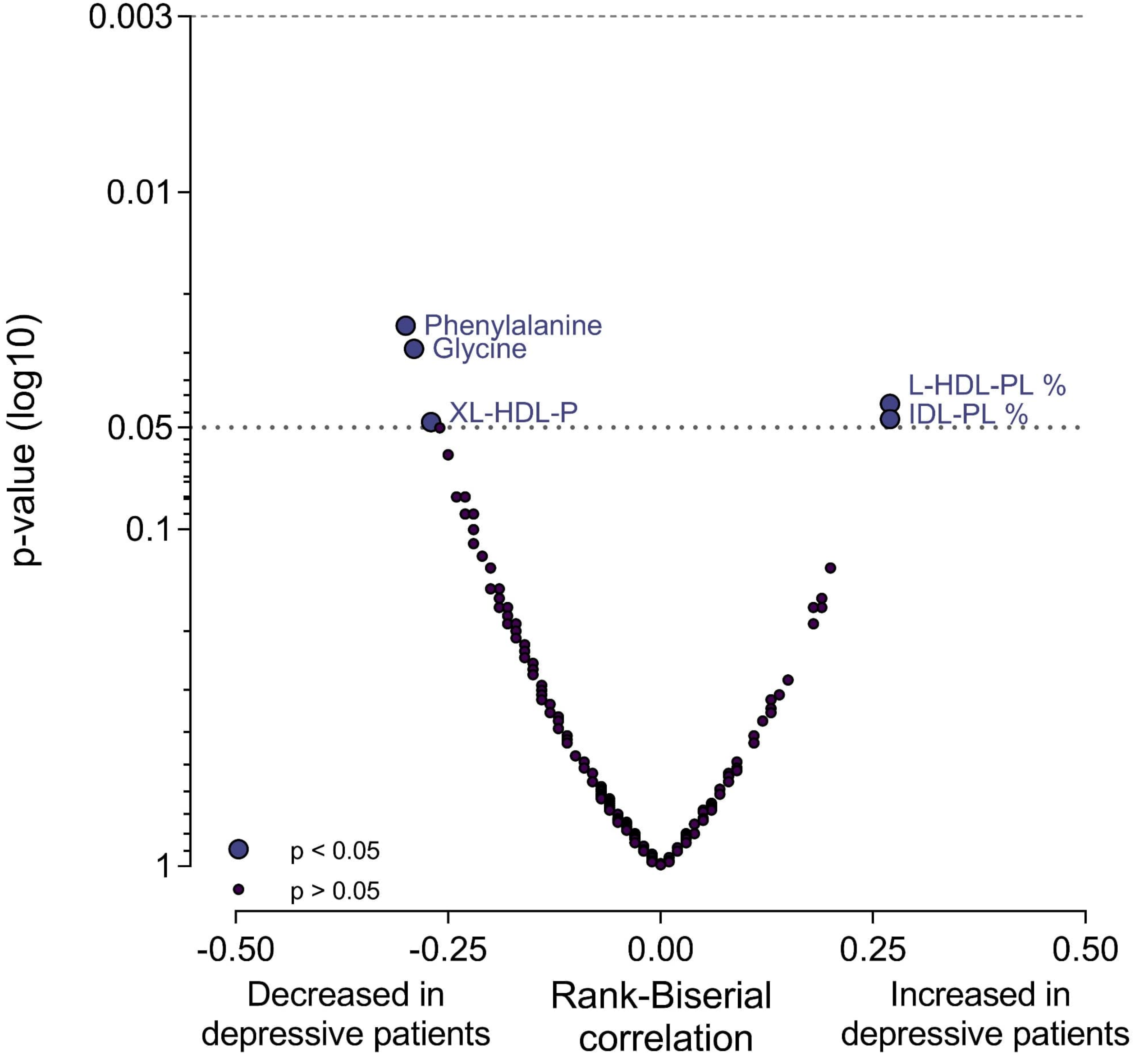

Fig S6 Volcano plot summarizing the impact of depression on the metabolic variables at delivery timepoint. The significance and magnitude of the observed changes between individuals suffering depression (n =62) and other mental health disorder (n = 28). Wilcoxon rank-sum test was used to compare the variables between the subgroups within the SSRI group. The effect is represented as rank-biserial correlation. XL-HDL-P, Concentration of very large HDL particles; L-HDL-PL %, Phospholipids to total lipids ratio in large high-density lipoprotein; IDL-PL %, Phospholipids to total lipids ratio in IDL
